# Conduction and validation of a novel mitotic spindle assembly related signature in hepatocellular carcinoma: prognostic prediction, tumor immune microenvironment and drug susceptibility

**DOI:** 10.3389/fgene.2024.1412303

**Published:** 2024-07-19

**Authors:** Zhao Zhang, Yuezhou Zhang, Gangli Hu, Qianxue Wu, Yang Zhou, Fang Luo

**Affiliations:** ^1^ Department of Hepatobiliary Surgery, The First Affiliated Hospital of Chongqing Medical University, Chongqing, China; ^2^ Central Laboratory, The First Affiliated Hospital of Chongqing Medical University, Chongqing, China; ^3^ Department of Hepatobiliary Surgery, The Second Affiliated Hospital of Chongqing Medical University, Chongqing, China

**Keywords:** HCC, nomogram, SAC3D1, mitotic spindle assembly, PI3K/Akt

## Abstract

**Introduction:** We have developed a risk-scoring model using gene expression levels related to mitotic spindle assembly (MSA) to predict the prognosis of liver cancer.

**Methods and results:** Initially, we identified 470 genes related to MSA from public databases. Subsequently, through analysis of sequencing data from liver cancer patient samples in online databases, we identified 7 genes suitable for constructing the risk-scoring model. We validated the predictive accuracy and clinical utility of the model. Through drug sensitivity analysis, we identified SAC3D1 as a gene sensitive to the most common anti-tumor drugs among these 7 genes. We propose SAC3D1 as a significant target for future clinical treatment. Furthermore, we conducted *in vivo* and *in vitro* experiments to validate the relevance of SAC3D1 to MSA and found its significant impact on the PI3K/Akt signaling pathway and spindle function.

**Conclusion:** Our research introduces a novel risk-scoring model that accurately predicts liver cancer prognosis. Additionally, our findings suggest SAC3D1 as a promising therapeutic target for hepatocellular carcinoma, potentially revealing new mechanisms underlying liver cancer development.

## Introduction

Hepatocellular carcinoma (HCC) ranks among the most prevalent liver tumors (Siegel et al., 2024) and globally stands as the fifth leading cause of cancer-related mortality (Wang and Deng, 2023). Despite advancements in HCC treatment and management (Wang et al., 2019), the 5-year survival rate remains dismally low (Wang et al., 2019), and the 5-year survival rate remains dismally low (Gravitz, 2014). The formidable challenge lies in the insidious and heterogeneous nature of liver cancer, complicating the identification of effective treatment targets (Deng et al., 2023). Moreover, the rapid metastasis common in most liver cancers (Li et al., 2021) often impedes complete surgical resection of tumor tissue (Maki and Hasegawa, 2022), with existing drugs prone to resistance development (Tang et al., 2020). Hence, the pursuit of new treatment targets becomes imperative.

It is widely recognized that cell proliferation relies on mitosis. During this process, a cell divides into two daughter cells while preserving the same genetic information. Errors occurring during mitosis, such as misalignment, can result in the generation of a substantial number of non-viable cells, often constituting a critical step in tumorigenesis (Tang et al., 2020). The assembly of the spindle apparatus plays a pivotal role during mitosis (Wittmann et al., 2001), and aberrant regulation of genes involved in this process can potentially induce errors in the entire mitotic process, consequently leading to tumorigenesis (Petry, 2016). There is evidence suggesting an association between MSA-related genes and tumorigenesis (Neumayer et al., 2014). Nonetheless, there has been no study addressing the impact of MSA-related genes on liver cancer.

In this study, we initially identified genes related to spindle assembly using online databases. Subsequently, we selected seven MSA-related genes with substantial implications for liver cancer, drawing upon sequencing data from liver cancer samples in TCGA. We developed a risk-scoring model utilizing the expression levels of these seven genes and performed a comprehensive series of analyses to investigate the influence of MSA-related genes on HCC progression. Ultimately, we validated one of these MSA-related genes in HCC through both *in vivo* and *in vitro* experiments, delving deeper into the specific mechanisms underlying its effects.

**SCHEME 1:**
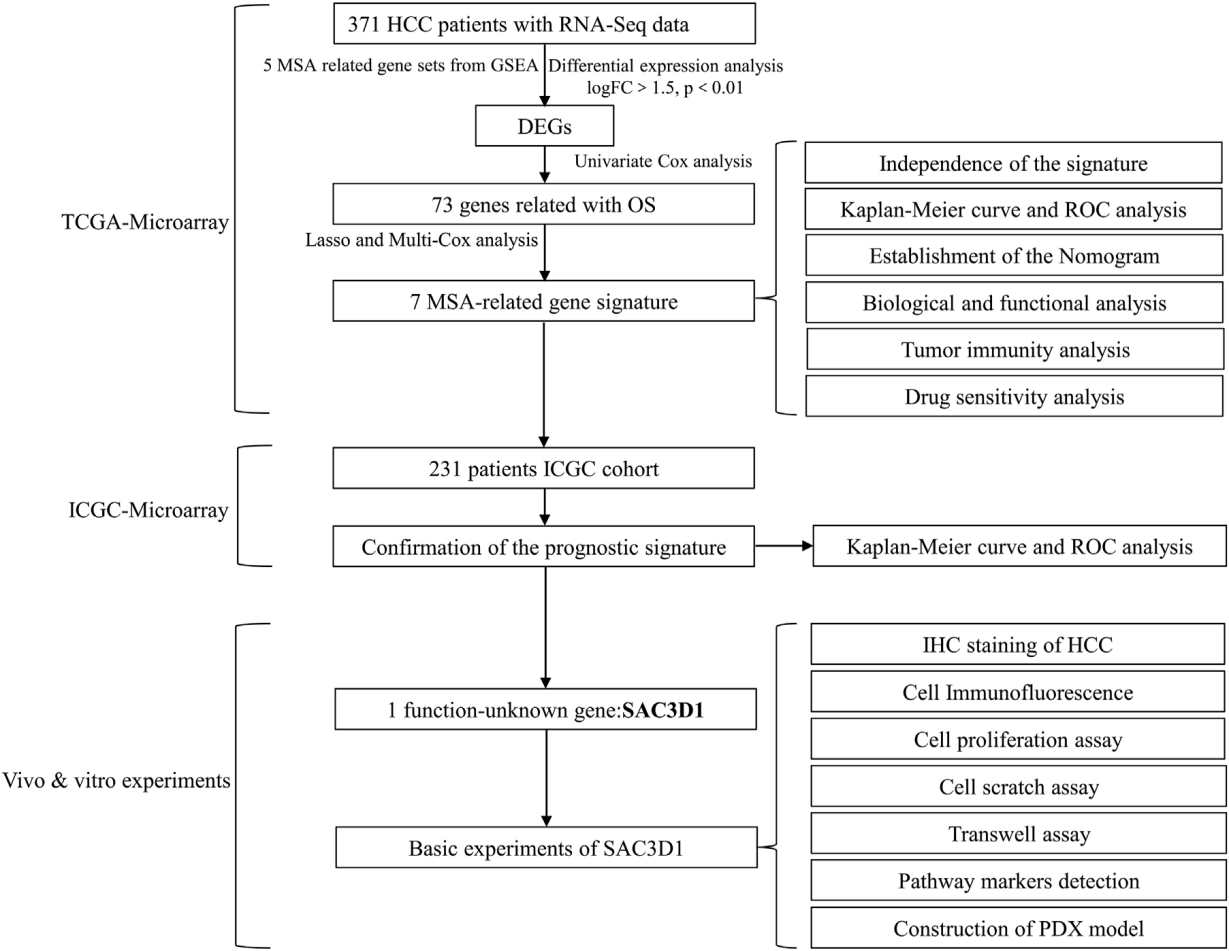
Workflow.

## Materials and methods

### MSA-related gene acquisition

We downloaded important gene sets related to MSA from the Molecular Signatures Database (https://www.gsea-msigdb.org/gsea/msigdb). The gene sets include HALLMARK MITOTIC SPINDLE (M5893), REACTOME MITOTIC SPINDLE CHECKPOINT (M27673), GOCC MITOTIC SPINDLE (M17132), GOBP REGULATION OF MITOTIC SPINDLE ASSEMBLY (M25022), and GOBP MITOTIC SPINDLE ASSEMBLY (M12435, M24697). After removing duplicates, a total of 470 MSA-related genes were filtered.

### Obtaining and organizing gene expression matrix and clinical data for hepatocellular carcinoma (HCC)

We obtained clinical data and transcriptome sequencing data of hepatocellular carcinoma patients from two different databases (Scheme 1). The data for liver cell hepatocellular carcinoma were downloaded from The Cancer Genome Atlas Program (TCGA, https://portal.gdc.cancer.gov; ID: LIHC, 2022), which included transcriptome and clinical data of 371 hepatocellular carcinoma (HCC) samples and 50 pairs of non-tumor liver tissue samples, to be used as the discovery cohort. Clinical pathological parameters included in the data are survival outcome, survival time, gender, age, AJCC stage, grade, serum alpha-fetoprotein (AFP) level, vascular invasion (VI), and Child-Pugh classification (Supplementary Material S1). Transcriptome and clinical data of LIRI-JP (n = 231) were extracted from the International Cancer Genome Consortium database (ICGC, https://dcc.icgc.org/projects; Code: LIRI-JP, 2022) and used as an external validation cohort. For batch effect removal, the necessary processing of the data was performed using the “limma” and “sva” packages.

### Constructing an MSA-related prognostic model

Based on genes related to MSA, univariate Cox regression was performed on the TCGA-derived liver cancer data to identify regulatory factors associated with overall survival (OS) in patients. Additionally, differential expression analysis was conducted to identify highly expressed mRNAs in hepatocellular carcinoma (criteria: logFC > 1.5, *p* < 0.01). Subsequently, Lasso regression and multivariate Cox regression analysis were applied to the intersecting gene data to reduce model covariance. Finally, a risk-scoring model for liver cancer patients was constructed using 7 MSA-related genes. The risk score for each sample was calculated using the following formula (Coef: risk coefficient; Exp: gene expression value; i: gene). Based on the obtained risk values, all patients were stratified into high and low-risk groups.

### Biological and functional analysis

We conducted an enrichment analysis of high-risk group genes using GSEA (version 4.3.3) to (Subramanian et al., 2005) understand the functions and pathways related to MSA. Gene sets were obtained from the MSigDB database, including Hallmark (h.all.v2023.2. Hs.symbols.gmt), Reactome (c2. cp.reactome.v2023.2. Hs.symbols.gmt), BioCarta (c2. cp.biocarta.v2023.2. Hs.symbols.gmt), PID (c2. cp.pid.v2023.2. Hs.symbols.gmt), and KEGG (c2. cp.kegg_legacy.v2023.2. Hs.symbols.gmt). Significant enrichment criteria were set as: |NES| > 1, NOM p-val < 0.05, and FDR q-val < 0.25.

### Establish a diagnostic score chart

We conducted univariate and multivariate Cox regression analysis using the “survival” package, through which we identified independent clinical risk variables and generated a forest plot. Subsequently, we integrated the risk score with independent prognostic features to enhance clinical analysis. Using the “rms” and “regplot” R packages, we plotted predictive diagnostic score charts for 1-year, 2-year, 3-year, 4-year, and 5-year survival rates of hepatocellular carcinoma. The effectiveness of this score chart was evaluated through calibration analysis.

### Clinical correlation analysis, immune correlation analysis, and tumor cell stemness analysis

To investigate the relationship between our risk score and clinical pathology, we created plots of predictive genes, risk scores, and clinical case factors using the survival and survminer R packages. Subsequently, to study the differences in immune correlation between the two risk groups, we evaluated the infiltration scores of 28 types of immune cells and immune-related pathways in different risk groups using the ssGSEA algorithm. Finally, we obtained a tumor stemness index calculated from mRNA expression and methylation signatures from previous studies. We then used the limma, ggplot2, ggpubr, and ggExtra R packages to assess the correlation between risk scores and tumor cell stemness.

### Drug sensitivity analysis

CellMiner is a comprehensive drug-related online resource that provides a wealth of drug data, including gene expression, mutation data, copy data, copy number variations, mutation data, and pharmacological response data. In this study, we identified drugs highly correlated with the expression levels of seven genes in the model using the CellMiner database (Supplementary Material S3).

### Cell culture

Human hepatocellular carcinoma cell lines (HCCLM3 and Huh-7) were obtained from the Chinese Academy of Sciences. HCC cells were cultured in DMEM (Cat No.: PM150210, Procell) containing 10% fetal bovine serum (FBS; Cat No.: 164210, Procell) and 1% penicillin/streptomycin (Cat. No.: 15140122, Thermo Scientific). These cell cultures were maintained at 37°C with a 5% CO_2_ atmosphere.

### Screening of knockdown sequences

Small interfering RNA (siRNA) oligonucleotides targeting SAC3D1 were designed and synthetized by Tsingke Biological Technology (China). SAC3D1-siRNA sequences were as follows:

si-SAC3D1-1: 5′-GCU​UCG​UGG​CAG​ACC​GCU​UTT-3′ (sense) and 5′-AAG​CGG​UCU​GCC​ACG​AAG​CTT-3′ (anti-sense); si-SAC3D1-2, 5′-GCA​GUG​CCA​UGU​GGG​CCA​UTT-3′ (sense) and 5′-AUG​GCC​CAC​AUG​GCA​CUG​CTT-3′ (anti-sense); si-SAC3D1-3, 5′-GGG​CCU​CUU​UCU​GCU​CUA​UTT-3′ (sense) and 5′-AUAGAGCAGAAAGAGGCCCTT′(anti-sense). Cells were transfected with 50 pmol siRNAs by the Lipofectamine 2000 (Invitrogen) for 6 h. Functional tests were performed at 3 days after transfection.

### Lentiviral transduction, and screening of stably transfected cell lines

The two HCC cell lines were passaged for four generations before undergoing lentiviral transduction using lentiviral particles from GenePharma, China. The cells were seeded in new six-well plates at a density of approximately 10%–15% (about 1.5 × 104 cells/well) until confluence reached around 20%, at which point the medium was replaced. The cells were then transduced with SAC3D1 interference lentivirus (shRNA) and control lentivirus at multiplicities of infection (MOI) of 10, 5, and 1, respectively, for HCC-LM3 and HuH-7 cells. After transduction, the cells were cultured in a medium containing puromycin (1 μg/mL, Cat. No.: A1113802, Thermo Scientific) for 3 days until no cell death was observed in the lentivirus-transduced groups under an inverted fluorescence microscope, with a transduction efficiency of approximately 100%. Stable SAC3D1-knockdown HCCLM3 and Huh-7 cell lines were established after more than five passages, during which the complete medium without puromycin was used. Real-time quantitative polymerase chain reaction (RT-PCR) and Western blotting were performed to assess changes in target gene expression. SAC3D1-shRNA sequences were as follows:si-SAC3D1: 5′-GCU​UCG​UGG​CAG​ACC​GCU​UTT-3′ (sense) 5′-AAG​CGG​UCU​GCC​ACG​AAG​CTT-3′ (anti-sense).

### RNA isolation and quantitative real-time PCR (qRT-PCR)

The RNA expression of the target genes (SAC3D1 and GAPDH) was quantified using a two-step reverse transcription-polymerase chain reaction (RT-PCR) analysis. Total RNA from stable SAC3D1-knockdown HCCLM3 and Huh-7 cell lines was extracted using a cell total RNA extraction kit (Cat. No.: RE-03111, FOREGENE). Subsequently, the RNA was converted to cDNA using a cDNA synthesis kit (Cat. No.: HY-K0510A, MCE). Quantitative real-time PCR was performed using a Bio-Rad CFX96 instrument with GAPDH as the reference gene. The following primer sequences were used for real-time PCR: SACD3D1 sense 5′-GCT​TCG​CTC​GTG​CCT​TTA​G-3′, antisense 5′-CGA​AGC​GAG​CAC​GGA​AAT​C-3′, GAPDH sense 5′-TGT​TGC​CAT​CAA​TGA​CCC​CTT-3′, antisense 5′-CTC​CAC​GAC​GTA​CTC​AGC​G-3′.

### Cell proliferation assay

The cell viability of both cells was determined using the Cell Counting Kit-8 (CCK-8, Order No. HY-K0301, MCE), which was performed according to the instructions provided by the manufacturer. Cells were seeded in 96-well plates with 100 μL of cell suspension per well (∼1,000 cells) and pre-incubated in a cell incubator for 4 days. 10 μL of CCK-8 solution was added to each well and the absorbance at 450 nm was measured using a microplate reader. Cell viability was calculated using the following formula: Cell viability = [(As-Ab)/(Ac-Ab)] × 100%, where: absorbance of experimental wells (containing treated cells, medium and CCK-8 solution); Ac: absorbance of control wells (containing control cells, medium and CCK-8 solution); Ab: absorbance of blank wells (containing medium and CCK-8 solution, without cells). Ab: absorbance of blank wells (with medium and CCK-8 solution, without cells).

### Clonogenic assay

The proliferative capacity of cells was assessed using the clonogenic assay. Log-phase control and treatment groups of liver cancer cells were taken, and a density of 400 cells per well was respectively seeded in new 6-well plates and cultured in DMEM medium containing 10% FBS. The medium was changed every 3 days, and the cells were cultured for 15 days until visible colonies appeared. Subsequently, the cells were gently washed once with PBS (Cat. No.: 10010023, Thermo Scientific), fixed with 4% paraformaldehyde solution (Cat. No.: P0099, Beyotime) for 15 min, stained with 0.1% crystal violet (Cat. No.: G1064, Solarbio), washed slowly with staining solution, and air-dried. The number of colonies (>50 cells) was then counted in each well under a microscope.

### Western blot

Prepare total cell protein lysate using RIPA buffer (Cat. No.: 89900, Thermo Scientific) supplemented with phosphatase inhibitor (Cat. No.: 78427, Thermo Scientific) and PMSF protease inhibitor (Cat. No.: 36978, Thermo Scientific). Then, perform SDS-polyacrylamide gel electrophoresis using gel (Cat. No.: PG112, Epizyme) and transfer to a polyvinylidene fluoride membrane (Cat. No.: IPVH00010, Merck). After membrane blocking with a rapid blocking buffer (Cat. No.: 37576, Thermo Scientific), incubate the membrane with primary antibodies SAC3D1 (Cat. No. 25857-1-AP, Proteintech, China) and GAPDH (Cat. No.: 10494-1-AP, Proteintech, China) overnight at 4°C. Subsequently, it was incubated with a secondary antibody (Cat No. GB23303, Servicebio), and finally detected by chemiluminescence using a chemiluminescent substrate kit (Cat. No.: 34580, Thermo Scientific).

### Scratch wound healing assay

The scratch wound healing assay is used to evaluate the migration ability of tumor cells. Log-phase liver cancer cells are seeded in a new 6-well plate and cultured overnight to form a dense monolayer of cells. After 24 h of serum starvation, a vertical scratch is made using a 10 µL pipette tip, followed by gentle washing with PBS three times. Subsequently, serum-free DMEM medium is added and photographs are taken. Another set of photographs is taken 48 h later. The initial scratch width is measured at *t* = 0, and the extent of migration is quantified as the percentage of closure of the scratch at *t* = 48 h relative to *t* = 0.

### Cell migration and invasion assay

The cell migration and invasion assay is used to assess the migration and invasion capabilities of cells. The experiment is conducted using small chambers (Corning) with polycarbonate membranes of 8 μm pore size. For the migration assay, 200 μL of a suspension containing log-phase tumor cells in serum-free culture medium is added to the upper chamber, while the lower chamber is filled with 600 μL of DMEM medium containing 10% FBS. After incubation at 37°C for 24 h, the cells are fixed with 4% paraformaldehyde for 20 min, the cells on the upper surface are removed, and the cells remaining on the lower surface are stained with 0.1% crystal violet, followed by photography and counting under an optical microscope.

### Tumorigenicity assay in BALB/c nude mice

Throughout the entire experimental process, strict adherence to animal experimental ethics was maintained, and approval was obtained from the Institutional Animal Care and Use Committee of Chongqing Medical University (IACUC-CQMU-2023-12046). A CDX model was established using 4-week-old male BALB/c nude mice. The mice were randomly divided into two groups (5 mice per group) and housed under standard conditions. After fixation and anesthesia, a suspension of log-phase NC-HCCLM3 and SH-HCCLM3 cells (5 × 10^6) was inoculated subcutaneously into the axillary region of each nude mouse. The volume of the implanted tumors was regularly measured and recorded. After 4 weeks, the mice were euthanized under ether anesthesia, and xenograft tumor tissues were collected for subsequent pathological examination and analysis.

### Immunofluorescence (IF)

The tissue samples were fixed in a 4% paraformaldehyde fixative solution (Cat. No: P0099, Beyotime), followed by embedding in paraffin and sectioning. Antigen retrieval was performed, and non-specific binding sites were blocked. The tissue sections were then incubated with a primary antibody specific for SAC3D1, followed by incubation with a secondary antibody conjugated with a fluorescent dye (Cat.No: GB21303, Servicebio). DAPI staining (Cat.No: C1005, Beyotime) was used for nuclear staining, and sealing with a specialized mounting medium. Immunofluorescence signals were observed using a fluorescence microscope (BX63, OLYMPUS), and image acquisition and analysis were performed.

### Immunohistochemistry (IHC)

Immunohistochemistry (IHC) was performed on paraffin-embedded samples following established protocols. Sections underwent de-waxing, rehydration through graded ethanol, and incubation in 3% hydrogen peroxide/methanol for 15 min to inhibit endogenous peroxidase activity. Antigen retrieval was achieved by boiling in a pressure cooker for 10 min with either citrate buffer (10 mM citrate, 0.05% Tween-20, pH 6) or Tris-EDTA buffer (1 mM Tris, 1 mM EDTA, 0.05% Tween-20, pH 9). After washing with PBS for 5 min, slides were blocked for 30 min using 10% normal goat serum (AR0009, Boster, China) or 1% BSA solution (19A15BO9, Boster, China). Primary antibodies (SAC3D1) were applied and incubated at 4°C overnight. Detection involved HRP-labeled secondary antibodies and DAB with hematoxylin counterstaining. Imaging was done using an Olympus BX63 microscope.

### Statistical analysis

Statistical analysis was performed using GraphPad Prism 9.0. Unless otherwise specified, data are presented as mean ± standard deviation (SD). Parametric tests were applied to normally distributed data, while non-parametric tests were used for non-normally distributed data. Multiple comparisons were made using parametric t-tests or one-way analysis of variance (ANOVA) followed by an LSD *post hoc* test to determine statistical differences. All statistical analyses were two-tailed, with a significance level set at *p* < 0.05.

## Result

### Establishment of an MSA-related risk score model

**FIGURE 1:**
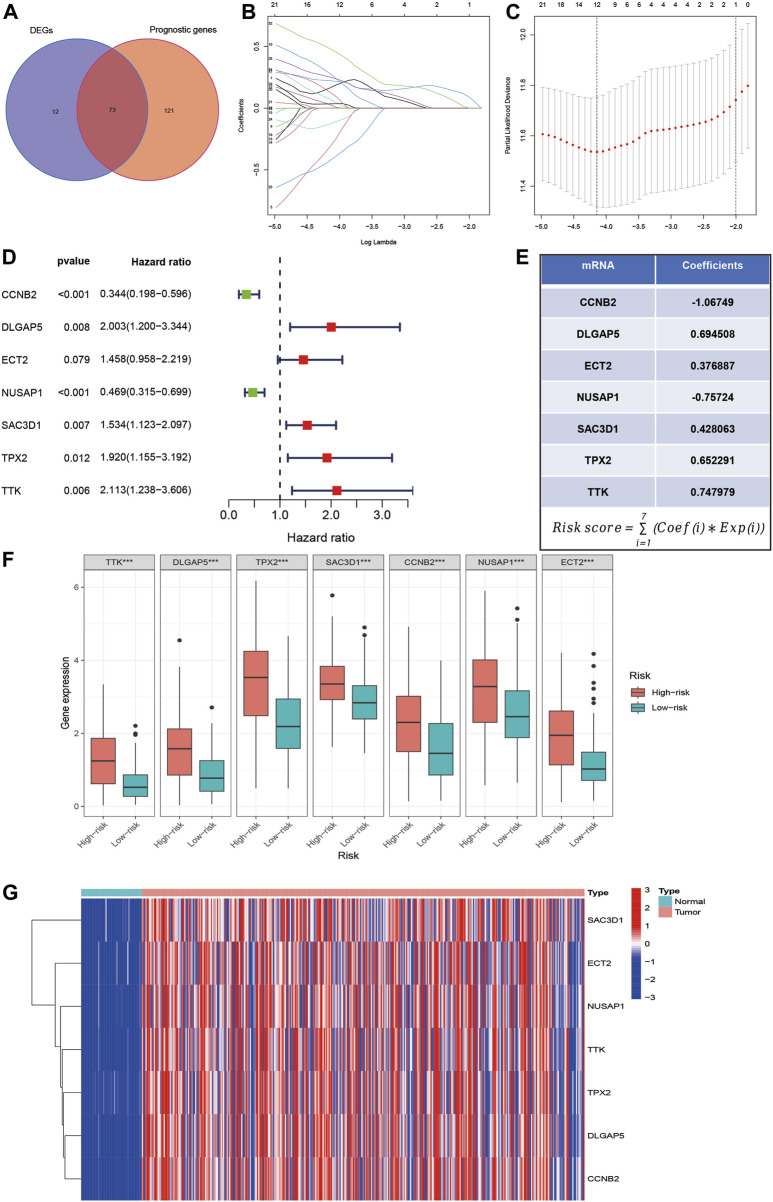
Selection of MSA-related genes associated with liver cancer prognosis and construction of a risk score model. **(A)** Intersection of MSA-related genes with differential expression in liver cancer samples and prognostic impact. **(B–D)** Identification of 7 MSA-related genes in liver cancer for constructing a prognostic risk score model through lasso regression and multivariate regression. **(E)** Formula used for calculating the risk score. **(F)** Expression levels of the 7 genes in high and low-risk groups. **(G)** Differential expression of these seven genes in tumor and normal samples.

We first downloaded 5 gene sets related to MSA from the MSigDB database. Combined with transcriptome sequencing data and clinical pathological data of HCC patients from the TCGA database, we obtained 73 MSA-related genes that showed differential expression and prognostic impact in HCC through Univariate regression analysis (Figure 1A). Furthermore, through Lasso regression analysis and Multivariate regression analysis, we selected 7 MSA-related genes (Figures 1B–D). Subsequently, we constructed a risk-scoring model for liver cancer patients, where the risk score for each sample was calculated using the formula in Figure 1E (Coef: risk coefficient; Exp: gene expression value; i: gene). Figure 1F displays the expression levels of the 7 genes in the high and low-risk groups, while Figure 1G presents a heatmap showing the differential expression of these seven genes in tumor samples and normal samples.

### The risk score model constructed based on the MSA has a diagnostic value

**FIGURE 2:**
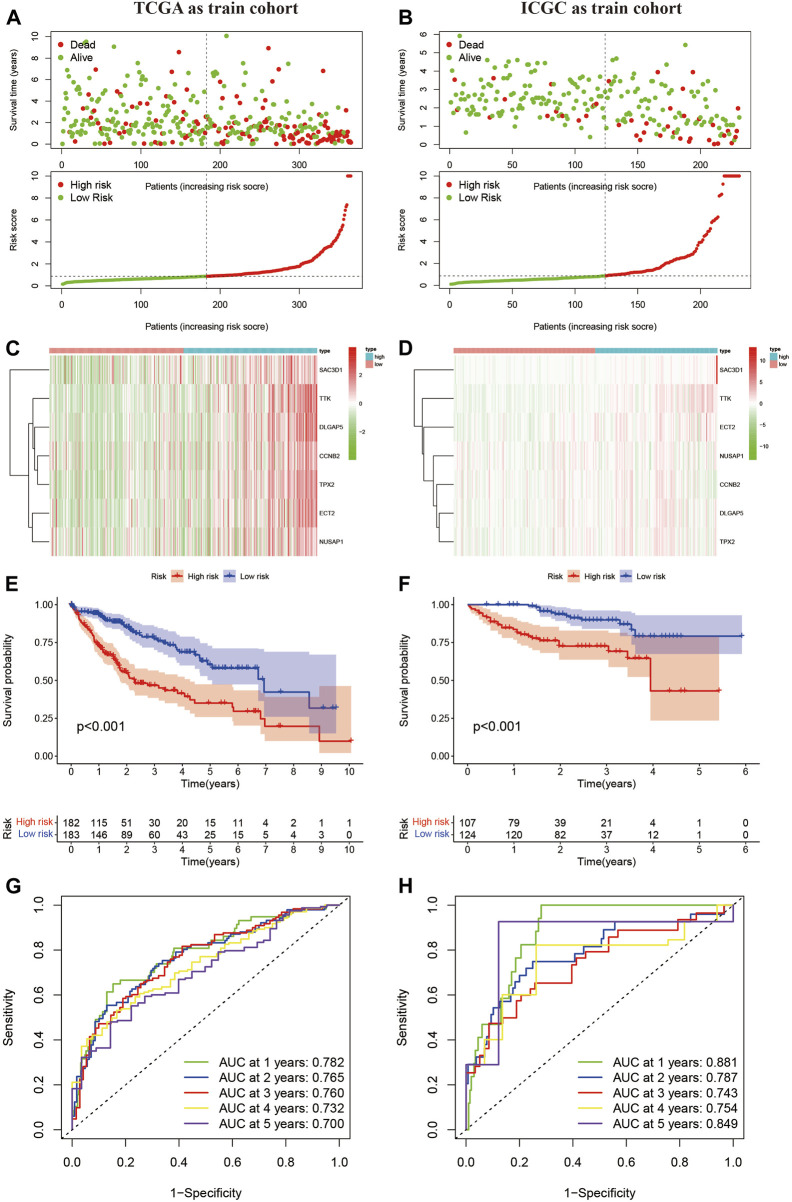
Confirmation of the constructed predictive features. **(A,B)** Liver cancer samples grouped based on the median risk score model. **(C,D)** Expression levels of 7 MSA-related genes in high and low-risk groups. **(E,F)** Prognostic curves for high and low-risk groups. **(G,H)** Time-dependent curves based on MSA, predicting overall survival (OS) at 1, 2, 3, 4, 5 years in TCGA and ICGC cohorts.

Next, we used the HCC sample data from the TCGA database as the training set and the HCC sample data from the ICGC database as the validation set. Based on the risk scoring model we constructed, we calculated the risk scores for each sample. Using the median risk score in the cohort as a reference, we categorized the samples from both datasets into high-risk and low-risk groups (Figures 2A, B). By plotting the heatmap, we observed that most samples in the high-risk group showed high expression of the 7 genes analyzed, while most samples in the low-risk group exhibited low expression (Figures 2C, D). Furthermore, we performed a Kaplan-Meier survival analysis on the samples from the training and validation sets. The results demonstrated that in both datasets, the high-risk group had a significantly worse prognosis compared to the low-risk group (Figures 2E, F). Finally, we evaluated the predictive ability of this classification using ROC analysis. According to the results shown in Figures 2G, H, the area under the curve (AUC) for 1, 2, 3, 4, and 5 years in the training set were 0.782, 0.765, 0.760, 0.732, and 0.700, respectively, all greater than 0.7. In the validation set, the AUC values for 1, 2, 3, 4, and 5 years were 0.881, 0.781, 0.743, 0.754, and 0.849, respectively, all greater than 0.7. These results indicate that the risk-scoring model we constructed based on MSA-related genes exhibits good sensitivity and specificity in predicting prognosis.

### The risk score is closely associated with tumor immunity

**FIGURE 3:**
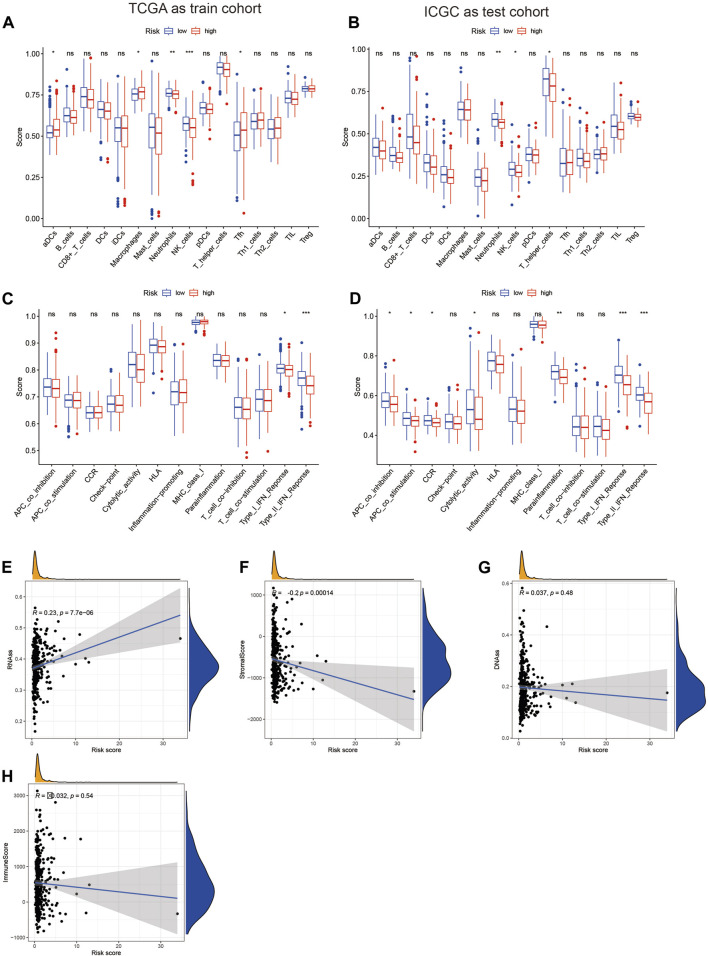
Analysis of the relationship between the risk score constructed based on 7 spindle assembly-related genes and tumor immunity and tumor stemness. **(A,B)** Display of expression differences of immune cells in high and low-risk groups. **(C,D)** Demonstration of differences in immune function in high and low-risk groups. **(E–H)** Relationship between high and low-risk groups and tumor stemness.

Previous studies have confirmed the important role of immune infiltration in HCC and its impact on patient prognosis (Yu et al., 2022). In our study, we used the TCGA database as the training set and the IGCG database as the validation set to score various immune cell infiltrations in the high-risk and low-risk groups. We found that neutrophils and NK cells had lower scores in the high-risk group, both in the training and validation sets (Figures 3A, B). Furthermore, we assessed the immune function between the high-risk and low-risk groups and found significant differences in the type I interferon response, with higher scores observed in the low-risk group in both the validation and training sets (Figures 3C, D). Finally, we conducted a tumor cell stemness analysis and found a positive correlation between the high-risk group and tumor stemness based on the RNAss score (Figure 3E). However, the other three scores showed negative correlations or were not significantly correlated in the high-risk group (Figures 3F–H). These results suggest that the high-risk group with elevated expression of seven MSA-related genes has a certain influence on tumor immune infiltration, but further discussion is needed regarding its impact on tumor stemness.

### The risk score can also predict the prognosis in liver cancer patients across different subgroups

**FIGURE 4:**
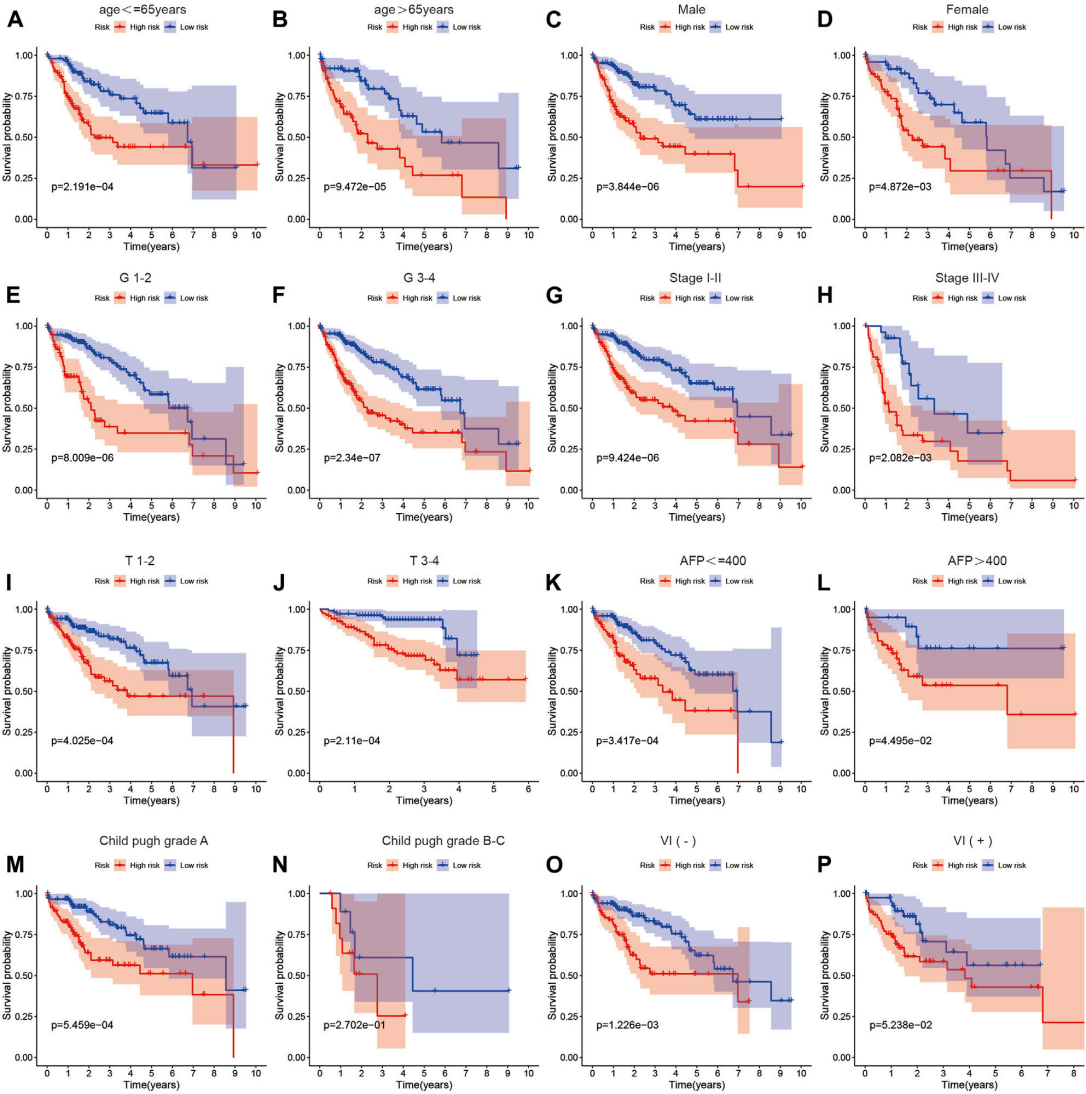
The relationship between the risk score and prognosis across different clinical factor strata. **(A–P)** Survival analysis results of high and low-risk groups in the risk score model among patients with different clinical characteristics.

To further validate the predictive ability of the risk score model constructed based on MSA-related genes for the survival of HCC patients, we subgrouped common clinical parameters of HCC patients and performed survival analysis. As shown in Figures 4A–P, in all subgroups including age>65 years, age ≤ 65 years, male, female, G1-2, G3-4, stage I-II, stage III-IV, T1-2, T3-4, AFP>400, AFP ≤ 400, Child-Pugh grade A, Child-Pugh grade B, C, and VI(−), VI(+), the high-risk group exhibited poorer survival. This further confirms the accurate predictive ability of our constructed model for the survival of HCC patients.

### The risk score constructed based on MSA can serve as an independent prognostic factor

**FIGURE 5:**
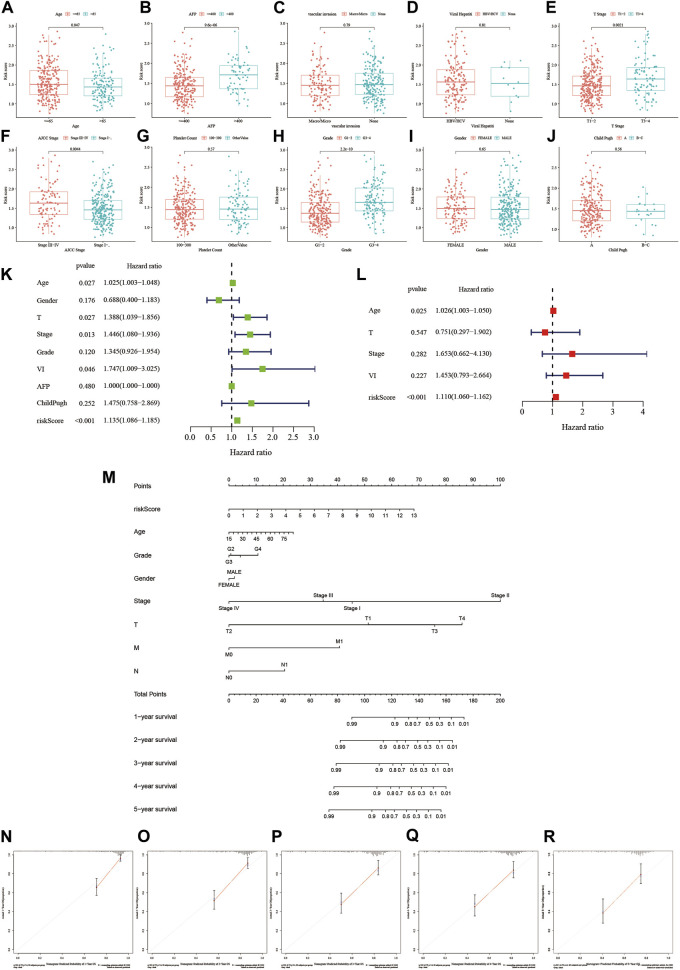
The relationship between the risk score and clinical factors and establishment of a nomogram. **(A–J)** The relationship between the risk score and different clinical features. **(K,L)** Investigating whether the risk score is an independent predictive factor through univariate and multivariate regression analysis. **(M)** Integrating the risk score with other clinical factors to create a scoring table. **(N–R)** Calibration plots: Validating the predictive accuracy of the diagnostic scoring table.

Further analysis of the correlation between our risk scoring model and common clinical pathological parameters revealed that most patients in the high-risk group were under the age of 65, had AFP levels >400, were at stage 3–4 in terms of T classification, and were graded at stage 3 or 4 according to AJCC staging (Figures 5A–J). This indicates a relationship between this risk scoring and the progression of HCC. To evaluate whether our risk scoring could serve as an independent risk factor for HCC, we conducted both univariate and multivariate regression analyses on the data. Based on the results of the univariate analysis shown in Figure 5K, we found that our risk scoring, along with age, stage, and vascular invasion, could predict patient prognosis (HR = 1.135, *p* < 0.001). Subsequently, through multivariate Cox analysis, we found that our risk-scoring model could serve as an independent prognostic indicator (HR = 1.11, *p* < 0.001) (Figure 5L). Additionally, we constructed a nomogram using risk scoring, age, stage, and other factors to predict the survival of HCC patients at 1, 2, 3, 4, and 5 years (Figures 5M–R). Validation showed good consistency between our predicted outcomes at 1, 2, 3, 4, and 5 years and the actual prognosis of patients.

### The MSA gene SAC3D1 is sensitive to most cancer drugs and affects the prognosis of liver cancer

**FIGURE 6:**
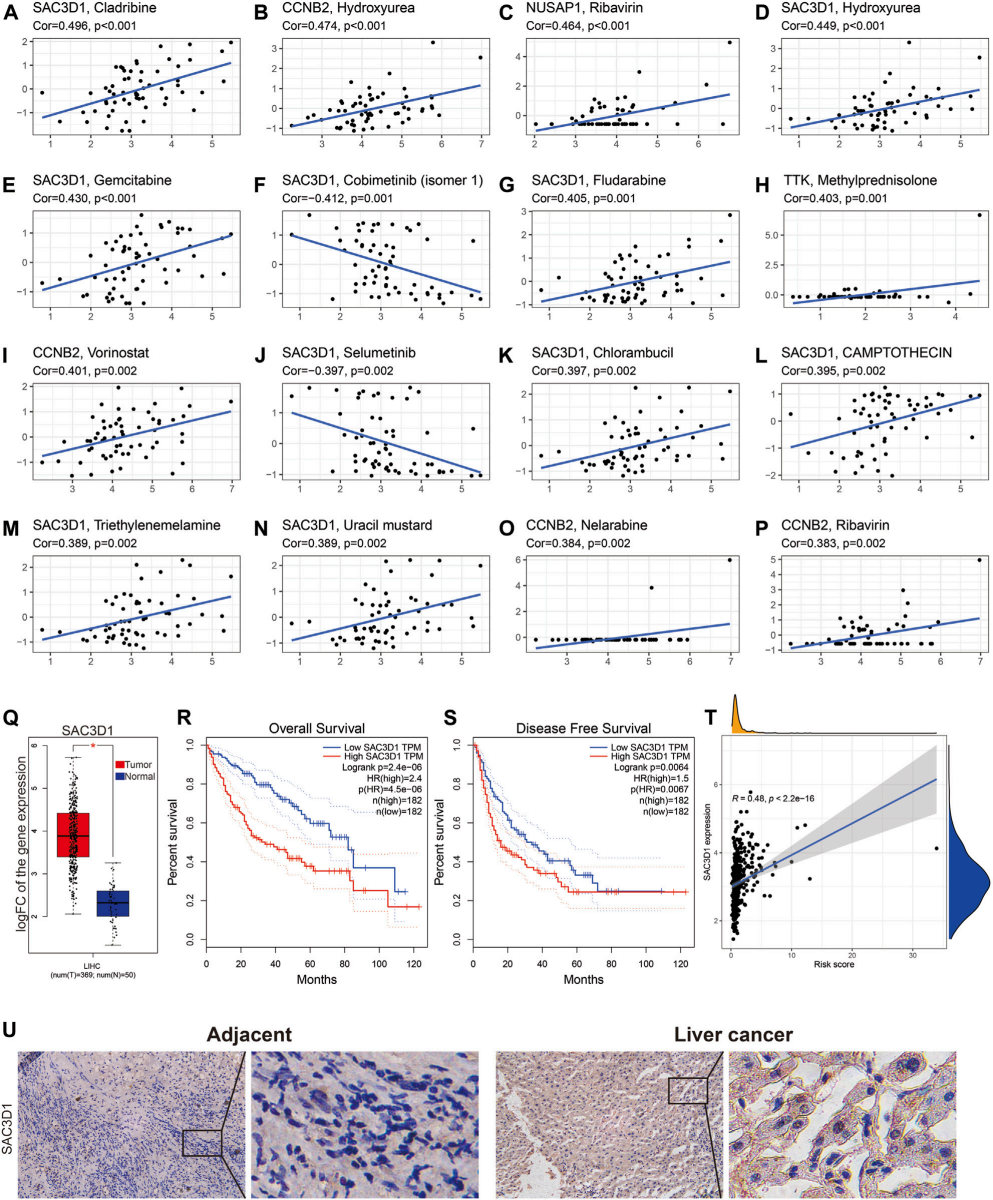
The relationship between SAC3D1 and cancer-related drugs and its analysis in TCGA liver cancer samples. **(A–P)** Relationship between SAC3D1 and common cancer drugs. **(Q)** Differential expression of SAC3D1 in liver cancer samples compared to normal samples. **(R–T)** The impact of SAC3D1 on OS and DFS of liver cancer patients. **(T)** Relationship between SAC3D1 and the constructed risk score. **(U)** The expression of SAC3D1 in hepatocellular carcinoma (HCC) tissues and adjacent non-cancerous tissues.

Next, based on the CellMiner database analysis, we identified 14 FDA-approved drugs that are most correlated with the expression of these 7 genes. We found a strong correlation between the expression of SAC3D1 and the sensitivity to 10 out of the 14 drugs. The sensitivity to drugs including Cladribine, Hydroxyurea, Gemcitabine, Fludarabine, Chlorambucil, Triethylenemelamine, and Uracil mustard was positively correlated with SAC3D1 expression, while the sensitivity to drugs including Cobimetinib and Selumetinib was negatively correlated with it (Figures 6A–P). Subsequently, through analysis of the SAC3D1 gene in the TCGA database, we observed its overexpression in tumors. High expression of SAC3D1 in liver cancer patients was associated with poor overall survival (OS) and recurrence-free survival (RFS). Furthermore, the expression level of this gene was positively correlated with the risk score we constructed. Similarly, after immunohistochemical staining of patient tumor tissues and adjacent non-cancerous tissues, we found that SAC3D1 was significantly overexpressed in the tumor tissues (Figures 6Q–U). Considering all these results, we conclude that the abnormally high expression of SAC3D1 is highly associated with the prognosis and drug treatment of patients with hepatocellular carcinoma (HCC).

**FIGURE 7:**
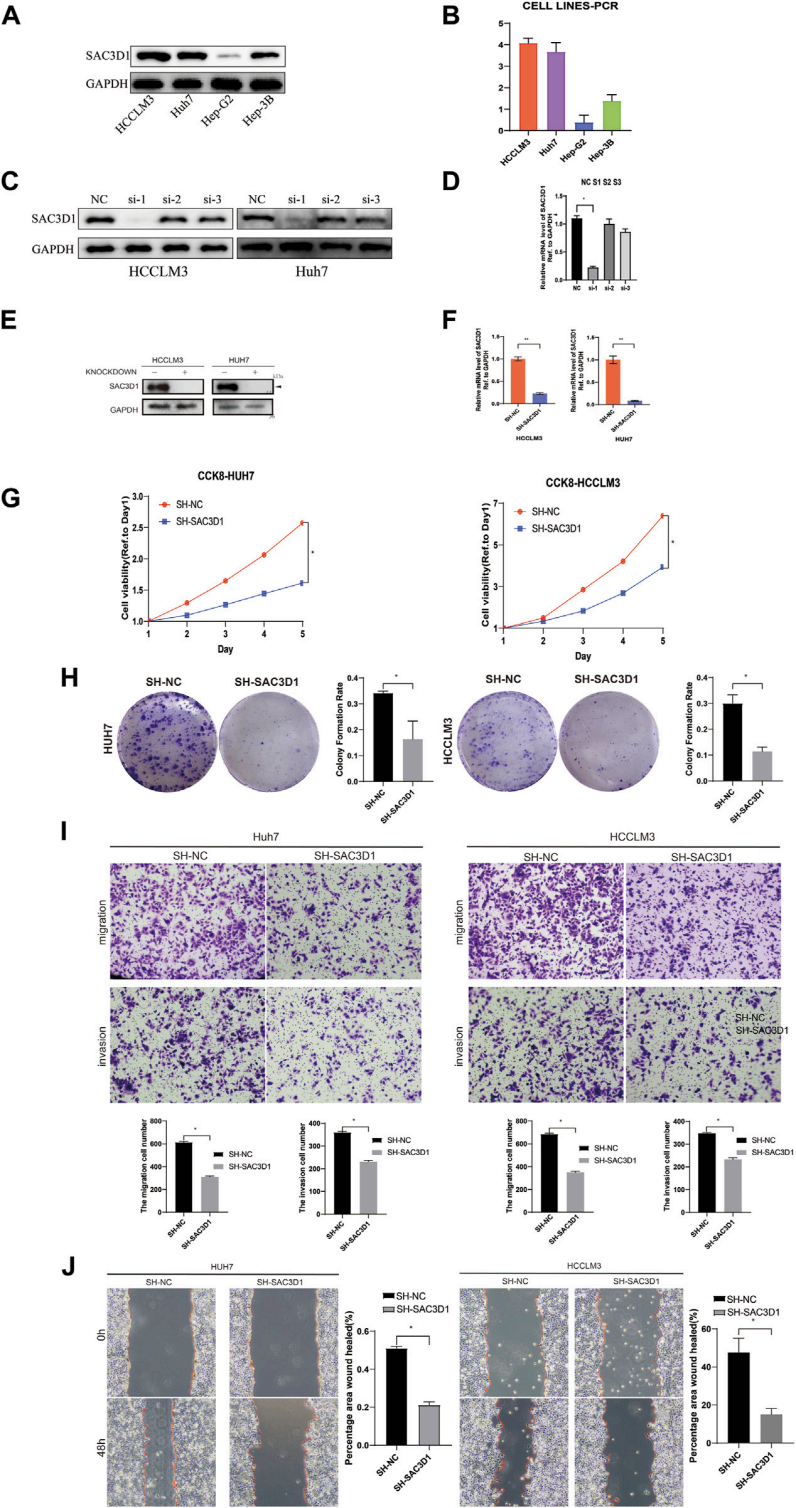
SAC3D1’s impact on liver cancer cells. **(A–D)** Cell line selection and knockdown sequences. **(E)** Validation of knockdown cells and control cells by Western blot. **(F)** Validation of knockdown cells by PCR. **(G)** Verification of cell proliferation by CCK-8 assay. **(H)** Verification of cell proliferation by colony formation assay. **(I)** Validation of cell migration and invasion capability by Transwell assay. **(J)** Scratch assay to verify cell proliferation.

To further validate the impact of SAC3D1 on hepatocellular carcinoma (HCC), we conducted *in vitro* experiments using two HCC cell lines, Huh7 and HCCLM3, expressing high levels of SAC3D1 (Figures 7A, B). Firstly, three different sequences were designed to knock down SAC3D1, and the most efficient knockdown sequence was selected through Western blot and qRT-PCR (Figures 7C, D). Subsequently, the selected sequence was used for lentiviral transfection, and the knockdown efficiency was confirmed through qRT-PCR and Western blot analysis (Figures 7E, F). A series of experiments were then performed, including CCK-8 assay (Figure 7G), colony formation assay (Figure 7H), Transwell assay (Figure 7I), and scratch assay (Figure 7J). The results of these experiments consistently demonstrated that in HCC cells with SAC3D1 knockdown, tumor growth and migration were weaker compared to the control group cells, indicating that the abnormal overexpression of SAC3D1 plays a significant promoting role in the proliferation and metastasis of HCC.

### The mechanism of action of SAC3D1 in influencing liver cancer development

**FIGURE 8:**
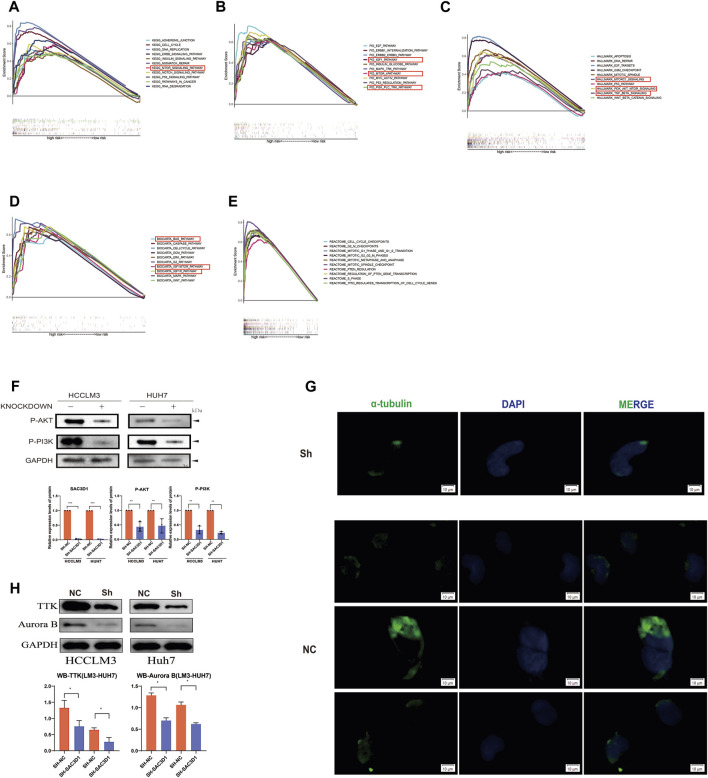
Validation of the mechanism by which SAC3D1 affects liver cancer. **(A–E)** Enrichment analysis using BIOCARTA, Hallmark, REACTOME, PID, and KEGG pathways to identify relevant pathways. **(F)** Validation of relevant pathways through Western blot. **(G)** Immunofluorescence staining for α-tubulin to detect spindle morphology in knocked-down cells compared to control cells. **(H)** Western blot was performed to detect the expression of TTK and Aurora proteins in both the knocked-down group cells and the control cells.

To investigate how SAC3D1 affects the progression of hepatocellular carcinoma (HCC), we conducted enrichment analysis of BIOCARTA, Hallmark, REACTOME, PID, and KEGG pathways in samples with high SAC3D1 expression using the GSEA database (Figures 8A–E). We identified multiple activated pathways, with the most significant being the PI3K/Akt signaling pathway. Furthermore, experimental validation through Western blotting confirmed a significant decrease in certain indicators of the PI3K/Akt signaling pathway in cells where SAC3D1 was knocked down (Figure 8F). These results suggest that SAC3D1 may impact tumor progression by affecting the PI3K/Akt signaling pathway. Additionally, through immunofluorescence staining, we found that compared to the control group, liver cancer cells with knocked-down SAC3D1 exhibited less spindle dysfunction: there was an increase in multipolar and monopolar spindle occurrences (Figure 8G). WB experiments similarly revealed a reduction in proteins representing spindle dysfunction, such as TTK and AuroraB, in the knockdown group (Figure 8H).

### Further evidence of the impact of SAC3D1 on liver cancer through *in vivo* experiments

**FIGURE 9:**
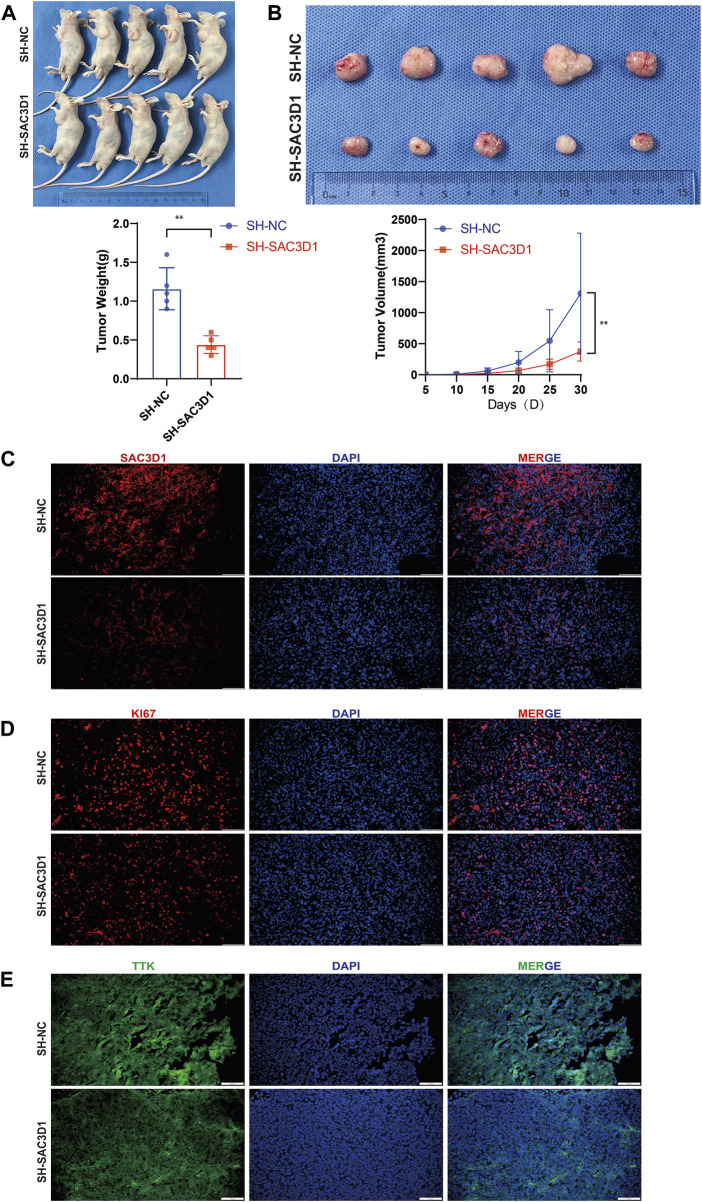
Analysis of the impact of SAC3D1 on liver cancer *in vivo*. **(A,B)** Demonstrate the effect of SAC3D1 on tumor growth rate. **(C)** Show differential expression of SAC3D1 in tumors with SACD31 knockdown compared to control group tumors. **(D)** Show differential expression of SAC3D1 in tumors with KI67 knockdown compared to control group tumors. **(E)** Immunofluorescence staining indicates that the expression of TTK protein is reduced *in vivo* in liver cancer cells with SAC3D1 knockdown.

To further validate whether SAC3D1 has an impact on the progression of hepatocellular carcinoma (HCC) *in vivo*, we conducted subcutaneous tumorigenesis experiments by injecting HCCLM3 cells with knocked-down SAC3D1 and control HCCLM3 cells into the subcutaneous tissue of nude mice on the same side of the armpit. Tumor dimensions were regularly measured to estimate volume, and new tumor tissues were collected 1 month later. We observed that tumors with knocked-down SAC3D1 exhibited significantly slower growth rates compared to the control group tumors (Figures 9A, B). Subsequently, immunofluorescence staining of the harvested tumor tissues revealed a markedly weaker expression of SAC3D1 in the knocked-down group compared to the control group (Figure 9C). Additionally, KI67 staining showed a significantly lower number of KI67-expressing cells in the tumors with reduced SAC3D1 expression compared to the control group tumors (Figure 9D), further confirming the significant impact of SAC3D1 on the proliferation of HCC *in vivo*. Research has found that TTK can accelerate the progression of tumors by promoting APC/C-CDC20-mediated mitosis in tumor cells (Chan et al., 2022). We discovered through immunofluorescence staining that the expression of TTK was reduced in tumor cells with knocked-down SAC3D1 (Figure 9E).

## Discussion

Liver cancer, especially hepatocellular carcinoma (HCC), ranks as the sixth most common cancer globally and the third leading cause of cancer-related mortality (Llovet et al., 2024). In China, HCC stands as the second highest among cancers causing mortality (Qi et al., 2023). In the pathogenesis of hepatocellular carcinoma (HCC), aberrant cell cycle regulation emerges as a notable characteristic, with the mitotic spindle playing a pivotal role in the cell cycle (Dominguez-Brauer et al., 2015). Comprising microtubules and associated proteins, the mitotic spindle forms a complex structure. Its principal function involves the accurate distribution of chromosomes during cell division, ensuring the precise segregation of genetic material into two daughter cells (Valdez et al., 2023). Normally, the proper assembly of the mitotic spindle facilitates precise chromosome segregation and regulates the rate of cell division and proliferation. However, abnormal assembly of the mitotic spindle can result in uncontrolled cell division and proliferation, leading to an increase in tumor cell numbers and tumor spread (Santaguida and Amon, 2015). Furthermore, abnormal assembly of the mitotic spindle may contribute to heightened genomic abnormalities and genetic heterogeneity in HCC cells. These genomic abnormalities may entail gene mutations, rearrangements, and alterations in gene copy numbers, potentially causing aberrant expression or dysfunction of crucial regulatory genes, thereby influencing the proliferation, metastasis, and drug resistance of HCC cells. Additionally, abnormal assembly of the mitotic spindle may impact the resistance of HCC cells to treatment. A dysfunctional mitotic spindle could lead to varied chromosomal and genomic characteristics in HCC cells, contributing to genetic heterogeneity that enhances resistance to conventional treatments such as chemotherapy or targeted therapy, consequently diminishing treatment efficacy and patient survival rates (Cohen-Sharir et al., 2021). Hence, comprehending the role of MSA in the onset and progression of HCC bears significant theoretical and clinical implications for unraveling the pathogenesis of HCC, identifying novel therapeutic targets, and guiding HCC treatment strategies.

In this study, we employed transcriptomic sequencing data and clinical pathological data from liver cancer (HCC) patients from TCGA and ICGC to comprehensively investigate the role of MSA-related genes in HCC prognosis and their potential therapeutic significance. Our research revealed several key findings: firstly, we identified a set of MSA-related genes significantly associated with HCC prognosis, including CCNB2, DLGAP5, ECT2, NUSAP1, SAC3D1, TPX2, and TTK. Through rigorous analytical methods such as univariate Cox regression, differential expression analysis, Lasso regression, and multivariate Cox regression, we established a robust model for prognostic risk scoring for HCC patients based on the TCGA cohort, with validation of the model’s predictive ability in the ICGC cohort. Kaplan-Meier survival analysis and ROC curve analysis demonstrated that the AUC values of our model in both cohorts were greater than 0.7 at 1, 2, 3, 4, and 5 years, indicating the strong predictive ability of our signature for the overall survival of liver cancer patients at these time points. Additionally, our model exhibited good discriminative power in various clinical subgroups, including age, gender, GRADE staging, STAGE grading, T staging, AFP, CHILD PUGH grade staging, and VI, enhancing its utility in diverse clinical settings.

Most HCCs are clonal and are believed to originate from cancer stem cells (Majumdar et al., 2012). The interaction between tumor cells and immune cells significantly influences patients’ response to immunotherapy and prognosis (Liu et al., 2023). Therefore, we explored the immune and tumor stem cell characteristics associated with the risk score of HCC patients. Our analysis revealed significant differences between the high-risk and low-risk groups in terms of immune cell infiltration and immune-related pathways. We found that neutrophils and NK cells had lower scores in the high-risk group in both the training and validation datasets. Evidence suggests that neutrophils can drive tumor progression through mechanisms such as immune evasion. Geh et al. (2022) propose that neutrophils play a central role in the pathogenesis of hepatocellular carcinoma (HCC) and have significant involvement in tumor initiation, local tumor progression, and metastasis. Natural killer (NK) cells, on the other hand, are distinctive cytotoxic lymphocytes that, akin to neutrophils, play a pivotal role in combating tumors (Sajid et al., 2022). These findings imply a potential association between the expression of MSA-related genes and the reshaping of the immune microenvironment in hepatocellular carcinoma (HCC). Moreover, our results suggest a positive correlation between the risk score and tumor stemness scores (RNAss and Stromal Score), underscoring the potential involvement of MSA-related genes in tumor initiation and progression. Utilizing the CellMiner database, we performed a drug sensitivity analysis to identify potential therapeutic targets among MSA-related genes. Our findings reveal a significant positive correlation between drug sensitivity and SAC3D1 expression for several drugs, including Cladribine, Hydroxyurea, CAMPTOTHECIN, Fludarabine, Gemcitabine, Chlorambucil, Triethylenemelamine, and Uracil mustard. Although some of these drugs may be utilized in liver cancer treatment, they are not typically the primary treatment option. For example, Gemcitabine, a metabolic inhibitor used to treat various solid tumors such as pancreatic and lung cancer, suppresses cancer cell proliferation by inhibiting DNA and RNA synthesis and may be employed as a first-line treatment for liver cancer, either as a monotherapy or in combination with other drugs (Jiang et al., 2022). CAMPTOTHECIN and Triethylenemelamine also exert their effects on DNA to impede cancer cell proliferation. Nevertheless, their utility is constrained by systemic toxicity, and they are generally not the preferred medications for treating liver cancer (Zhang et al., 2023). The remaining medications, such as Cladribine, Hydroxyurea, Fludarabine, and Chlorambucil, disrupt DNA synthesis to impede cancer cell proliferation and are primarily employed in the treatment of certain hematological malignancies (Niemann et al., 2023; Li et al., 2024). Similarly, Uracil mustard, an alkylating agent, has been phased out due to its severe side effects and resistance and is no longer clinically utilized for cancer treatment. Conversely, drug sensitivity, including Cobimetinib (isomer 1) and Selumetinib, is inversely correlated with them. For HCC patients with BRAF V600E mutations, the consideration of Cobimetinib (isomer 1) and Selumetinib for targeted therapy may be warranted.

Upon reviewing the literature, it is evident that CCNB2 expression in hepatocellular carcinoma (HCC) demonstrates a significant correlation with KPNA2, implicating its involvement in mitotic and cell cycle regulatory processes, thereby exerting a substantial impact on HCC progression (Gao et al., 2018). DLGAP5 has been identified as being upregulated across various cancer types, including hepatocellular carcinoma, bladder cell carcinoma, and migratory cell carcinoma. Elevated DLGAP5 expression correlates with diminished serum-dependent and anchorage-independent growth of cancer cells. DLGAP5 promotes non-small cell lung cancer (NSCLC) cell proliferation through modulation of the cell cycle, rather than apoptosis. Its involvement in cell-cycle regulation is characterized by the induction of microtubule-protein sheet formation and facilitation of spindle formation. Additionally, DLGAP5 undergoes phosphorylation by AURKA, consequently activating the NFκB signaling pathway and fostering oncogenesis through p53 degradation. Moreover, DLGAP5 regulates its nuclear localization and cyclin E1 expression via Aurora-A-mediated phosphorylation and interaction with NF-κB, thereby promoting G1 phase progression and cell proliferation (Wang et al., 2018). ECT2 facilitates early recurrence and metastasis of hepatocellular carcinoma through activation of the Rho/ERK signaling axis and interaction with RACGAP1. Elevated ECT2 expression significantly correlates with poorer patient survival (Chen et al., 2015). NUSAP1 serves as a pivotal oncogenic factor in pancreatic ductal adenocarcinoma, driving proliferation, migration, and epithelial-mesenchymal transition, while inhibiting the AMPK signaling pathway. Elevated NUSAP1 expression is associated with unfavorable prognosis (Liu et al., 2024). Furthermore, heightened expression of TPX2 in HCC promotes cell proliferation and invasion through AKT signaling activation and upregulation of MMP2/9, underscoring its potential as a therapeutic target for hepatocellular carcinoma (Liu et al., 2014). TTK inhibitors exhibit heightened sensitivity toward tumor cells harboring CTNNB1 mutations, implicating CTNNB1 mutations as predictive genetic markers for patient response to TTK inhibitor therapy (Zaman et al., 2017). Based on the aforementioned research advancements and drug sensitivity analyses, we have chosen SAC3D1, currently understudied in hepatocellular carcinoma, as the focus for subsequent *ex vivo* experiments.

SAC3D1 is a protein analogous to α-tubulin, localized to the centrosome during interphase and to the spindle and mitotic spindle during the M phase. Suppressing its expression can lead to defects in centrosome duplication and spindle formation, resulting in polyploidy and involvement in tumorigenesis (Khuda et al., 2004). Research conducted by Haitao Wang et al.(2018) has demonstrated that SAC3D1 can contribute to the progression of liver cancer by activating the Wnt/β-catenin signaling pathway (Wang and Shi, 2022). Studies by Liu et al. (2020) suggest that the upregulation of SAC3D1 expression is linked to the advancement of gastric cancer. The findings indicated prevalent SAC3D1 expression in human liver cancer tissues, with SAC3D1 expression augmenting the proliferation and invasion of liver cancer cells, suggesting its promotional role in HCC progression. To elucidate the potential molecular mechanisms underlying SAC3D1’s oncogenic role in HCC, pathway enrichment analysis revealed its involvement in activating the PI3K/Akt signaling pathway in HCC cell lines. Cellular immunofluorescence staining and Western blot assays of hepatocellular carcinoma cells in the SAC3D1 knockdown expression group exhibited reduced expression levels of Aurora B and TTK, implying that SAC3D1 knockdown mitigated SAC function dysregulation. This discovery delineates the downstream signaling pathway of SAC3D1 in inducing tumor progression in HCC. In conclusion, our study comprehensively elucidates the significant importance and functional impact of MSA-related genes in the prognosis of HCC. Furthermore, an effective prognostic risk-scoring model for liver cancer patients has been established. These findings not only contribute to comprehending the pathogenesis of HCC but also offer potential prognostic biomarkers and therapeutic targets for managing HCC.

Future research endeavors could enhance our comprehension of the intricate regulatory mechanisms of MSA in HCC by delving deeper into this process. Potential research methodologies may encompass utilizing cellular and animal models to simulate aberrant MSA and employing diverse molecular biology and cell biology techniques to elucidate the mechanisms underlying these aberrations. Moreover, drug screening experiments could be undertaken to identify compounds capable of rectifying abnormal MSA, thereby ameliorating the prognosis and treatment outcomes for HCC patients. Additionally, cutting-edge technologies such as single-cell transcriptomics can be harnessed to scrutinize the cellular heterogeneity of MSA abnormalities in liver cancer cells and their correlation with tumor development. These investigations will furnish crucial theoretical and experimental groundwork for devising precision therapeutic strategies targeting aberrant MSA.

## Data Availability

The original contributions presented in the study are included in the article/Supplementary Material, further inquiries can be directed to the corresponding author.
