# Supplementary figures and images for: Conduction and validation of a novel mitotic spindle assembly related signature in hepatocellular carcinoma: prognostic prediction, tumor immune microenvironment and drug susceptibility
Source: Front Genet. 2024 Jul 19;15:1412303. doi: 10.3389/fgene.2024.1412303 (PMC11294156; doi:10.3389/fgene.2024.1412303)

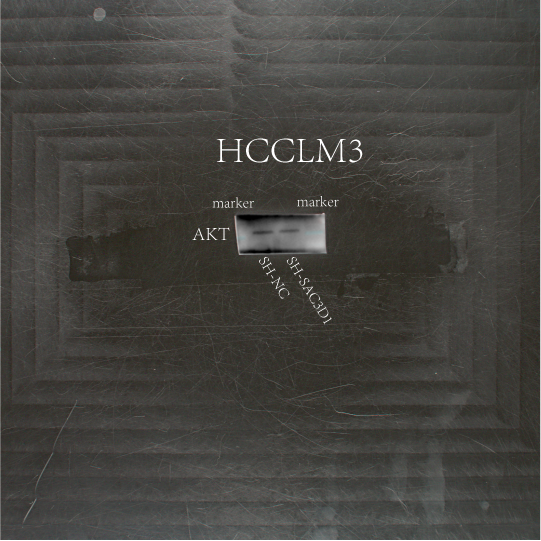

Supplement: Supplementary file 2 [file DataSheet2.ZIP › wb/AKT-HCCLM3-ú¿1ú⌐.tif]

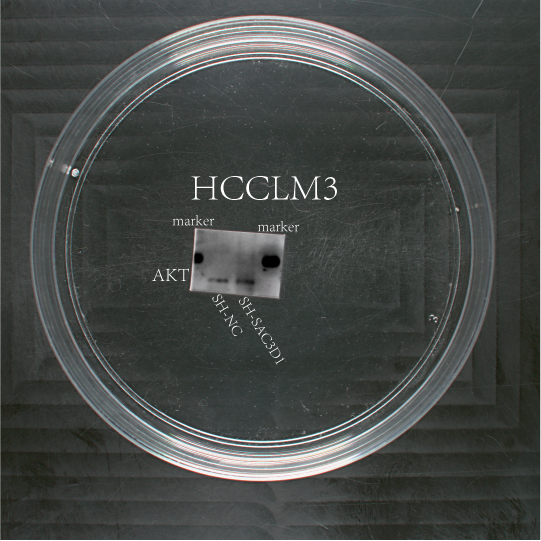

Supplement: Supplementary file 2 [file DataSheet2.ZIP › wb/AKT-HCCLM3-ú¿2ú⌐.tif]

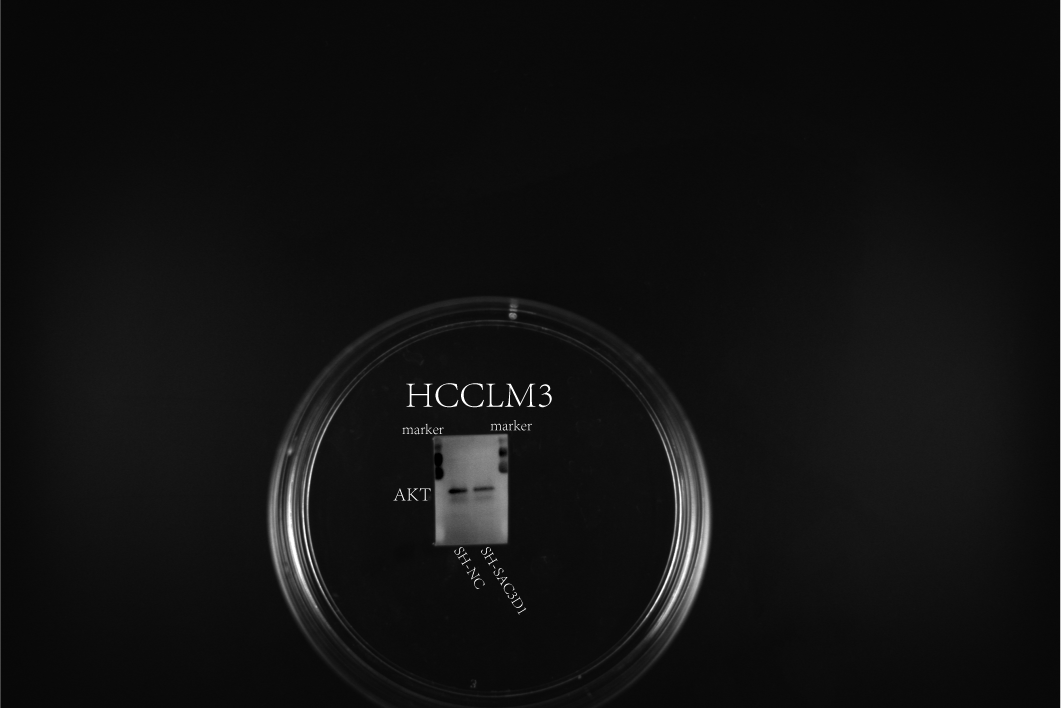

Supplement: Supplementary file 2 [file DataSheet2.ZIP › wb/AKT-HCCLM3-ú¿3ú⌐.tif]

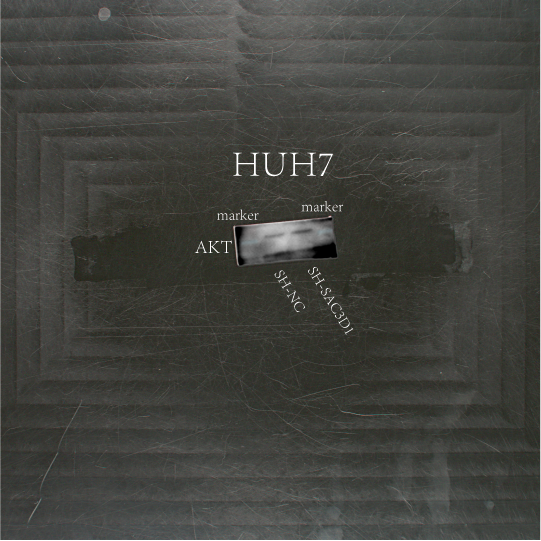

Supplement: Supplementary file 2 [file DataSheet2.ZIP › wb/AKT-HUH7-ú¿1ú⌐.tif]

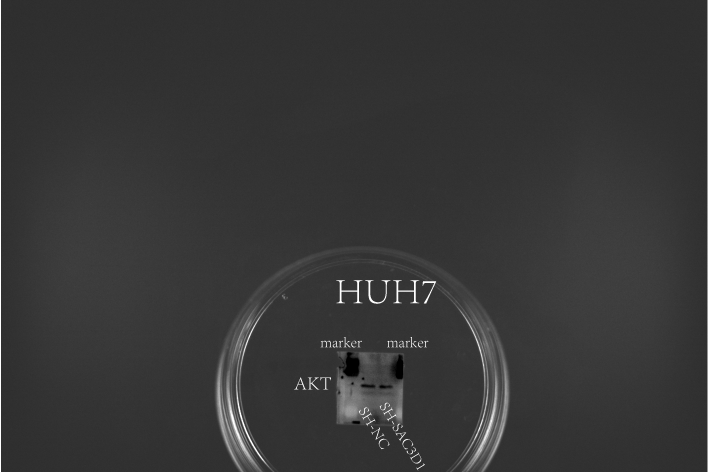

Supplement: Supplementary file 2 [file DataSheet2.ZIP › wb/AKT-HUH7-ú¿2ú⌐.tif]

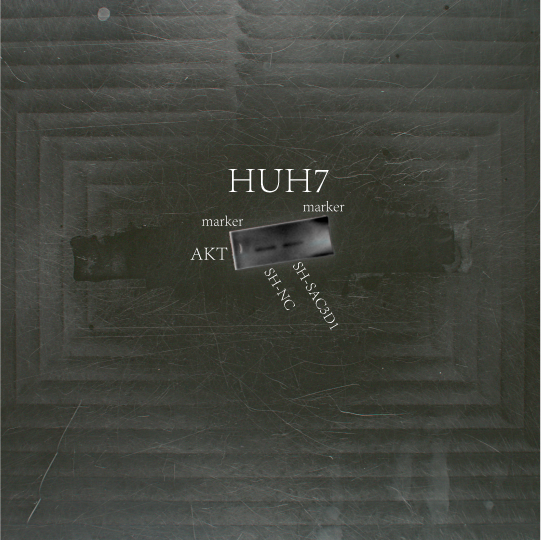

Supplement: Supplementary file 2 [file DataSheet2.ZIP › wb/AKT-HUH7-ú¿3ú⌐.tif]

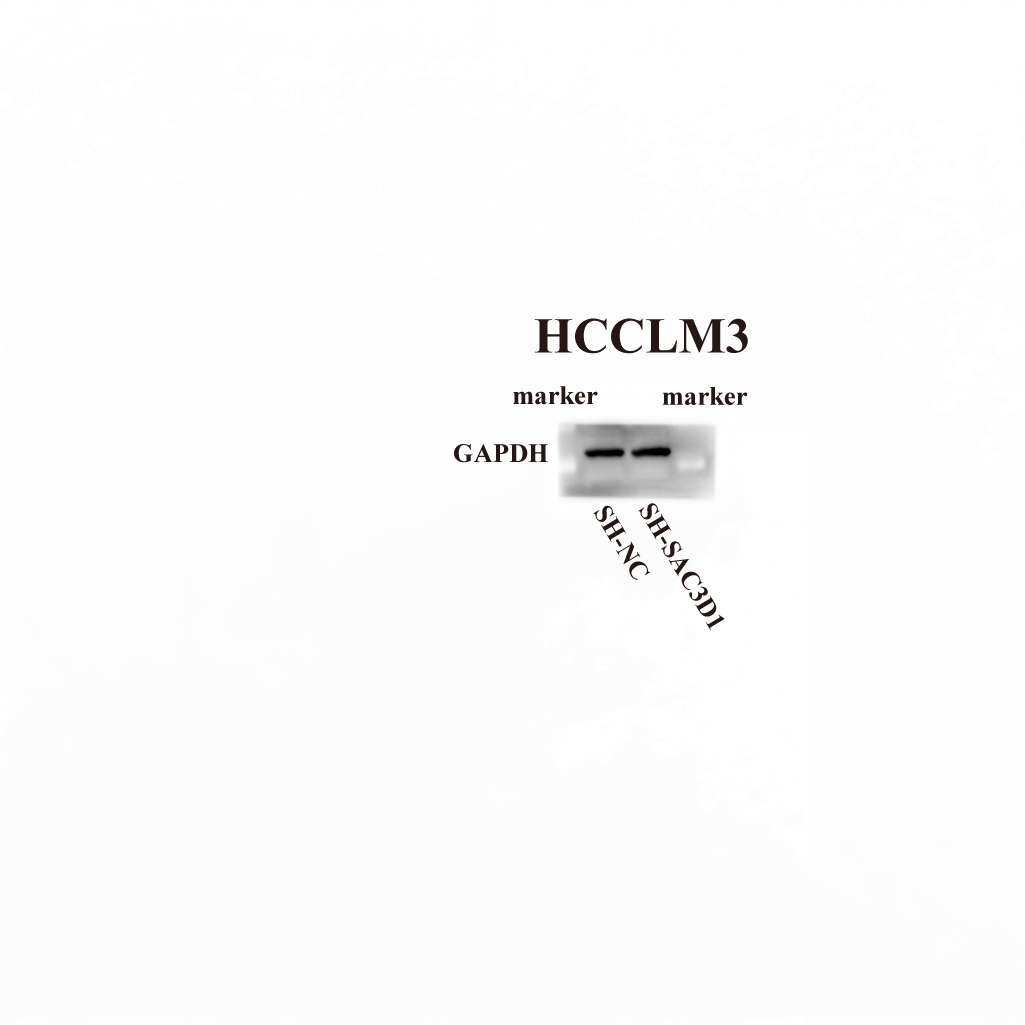

Supplement: Supplementary file 2 [file DataSheet2.ZIP › wb/GAPDH-HCCLM3-(1).tif]

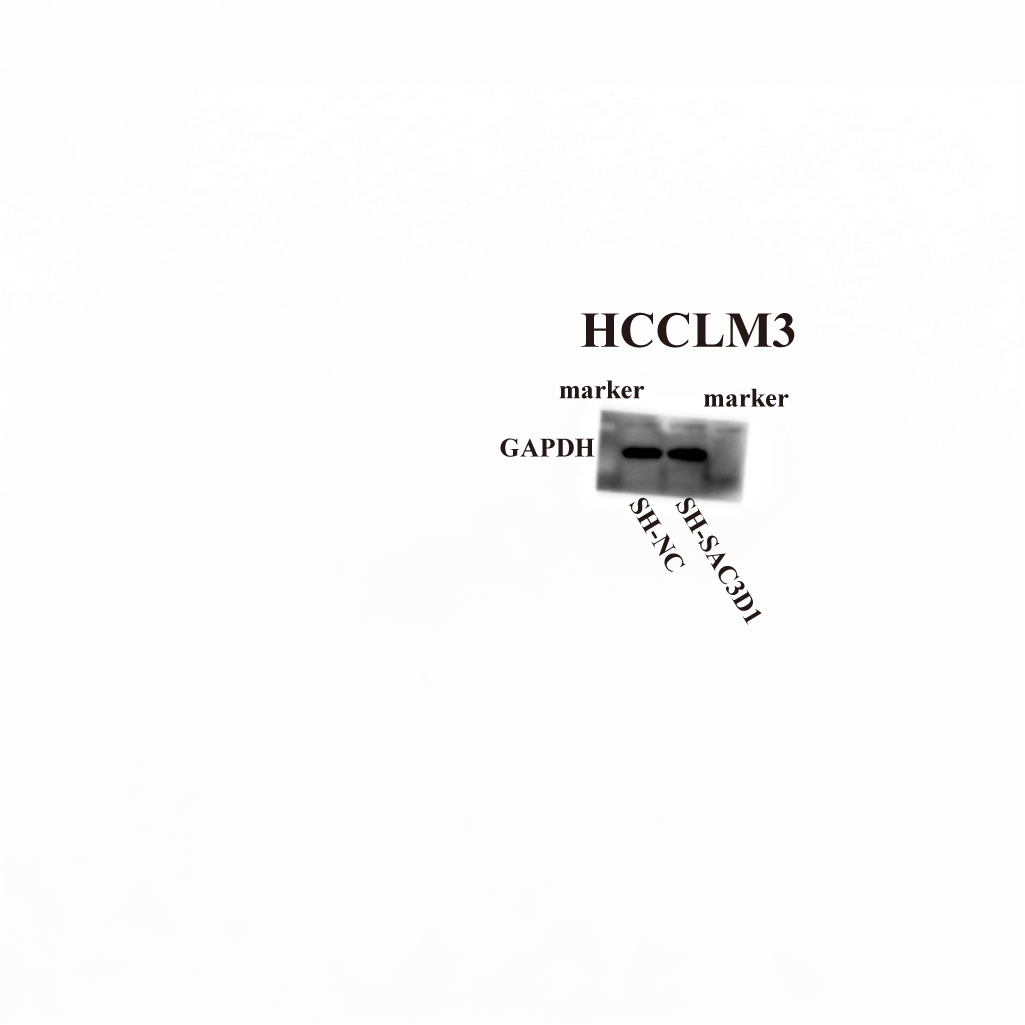

Supplement: Supplementary file 2 [file DataSheet2.ZIP › wb/GAPDH-HCCLM3-(2).tif]

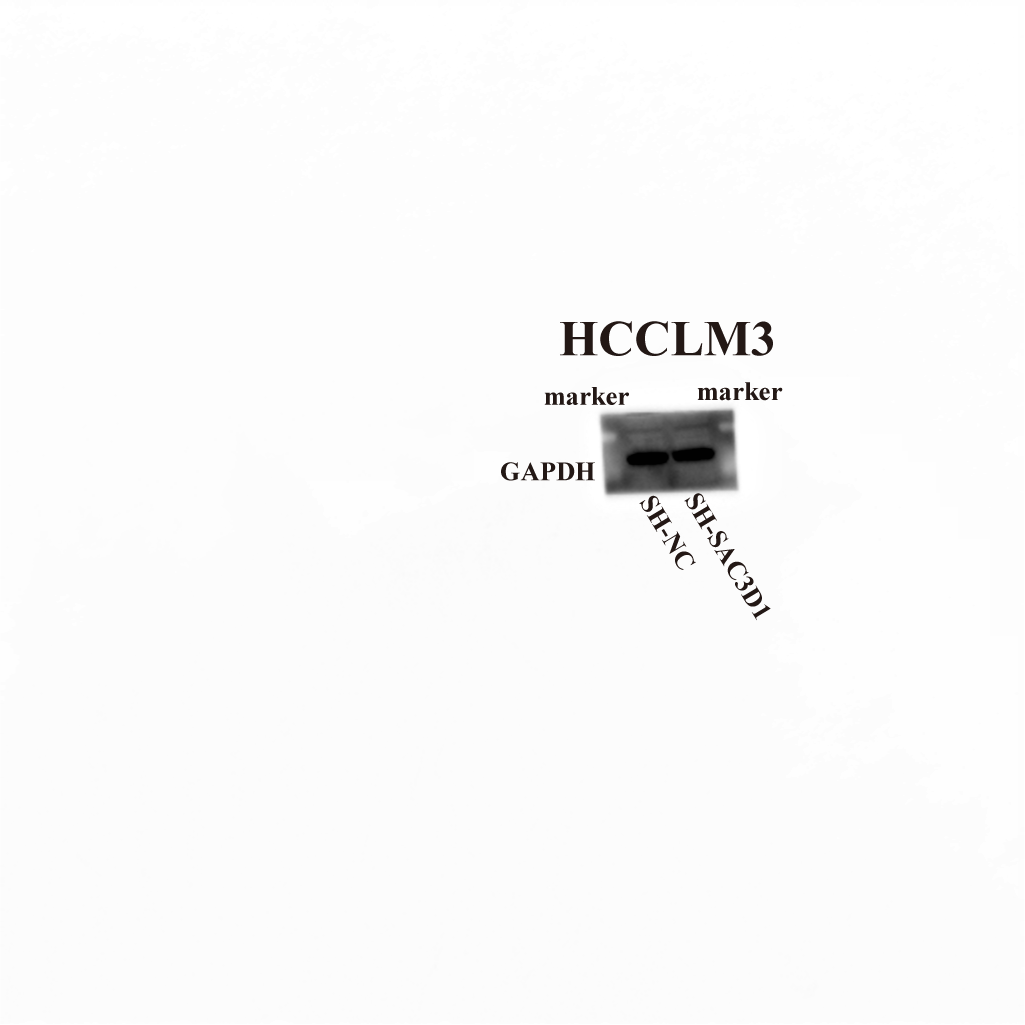

Supplement: Supplementary file 2 [file DataSheet2.ZIP › wb/GAPDH-HCCLM3-(3).tif]

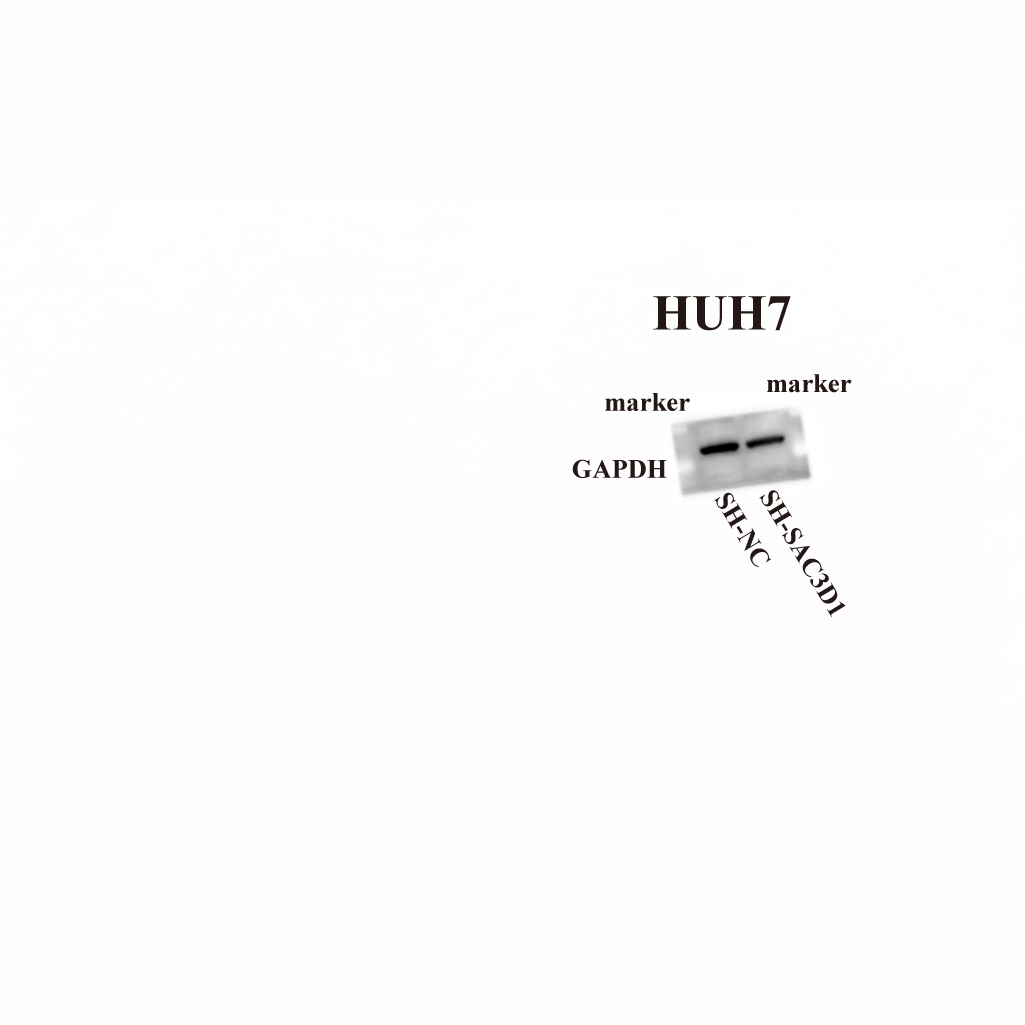

Supplement: Supplementary file 2 [file DataSheet2.ZIP › wb/GAPDH-HUH7-(1).tif]

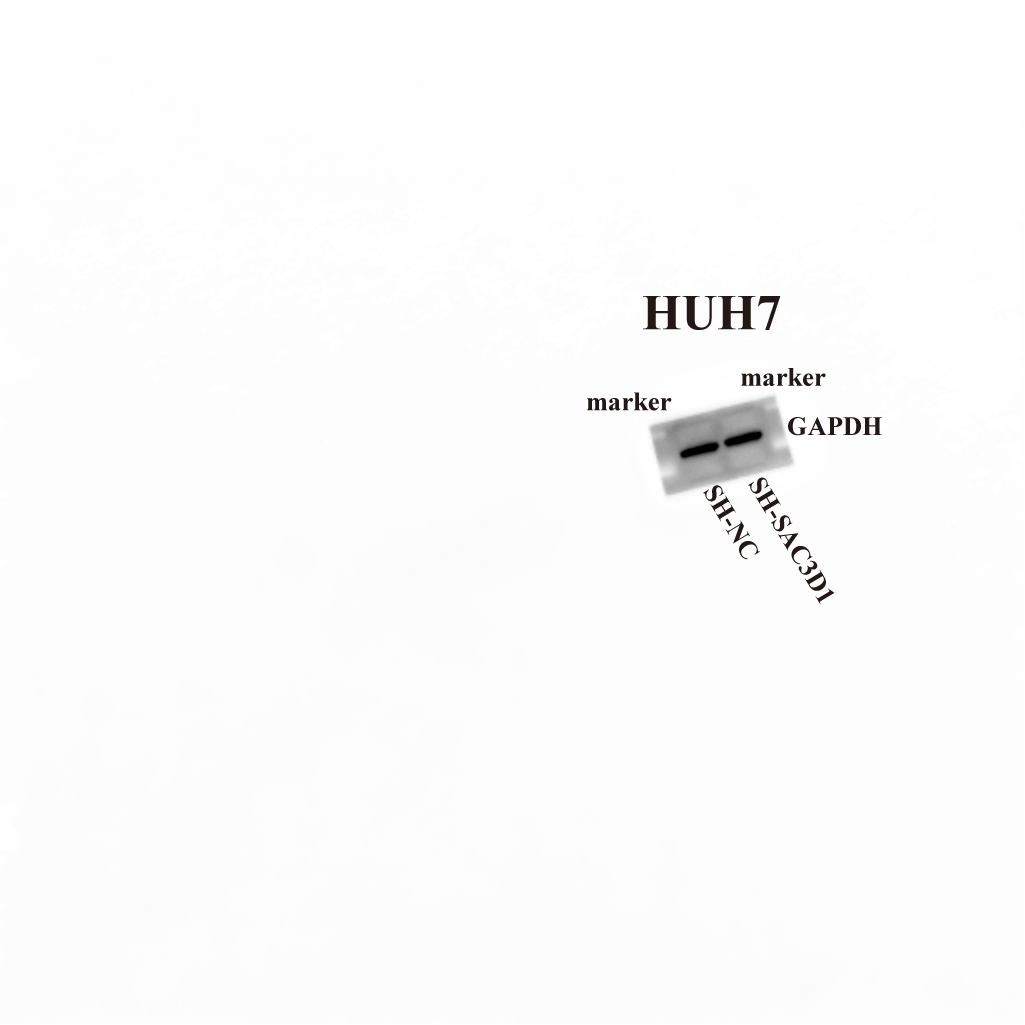

Supplement: Supplementary file 2 [file DataSheet2.ZIP › wb/GAPDH-HUH7-(2).tif]

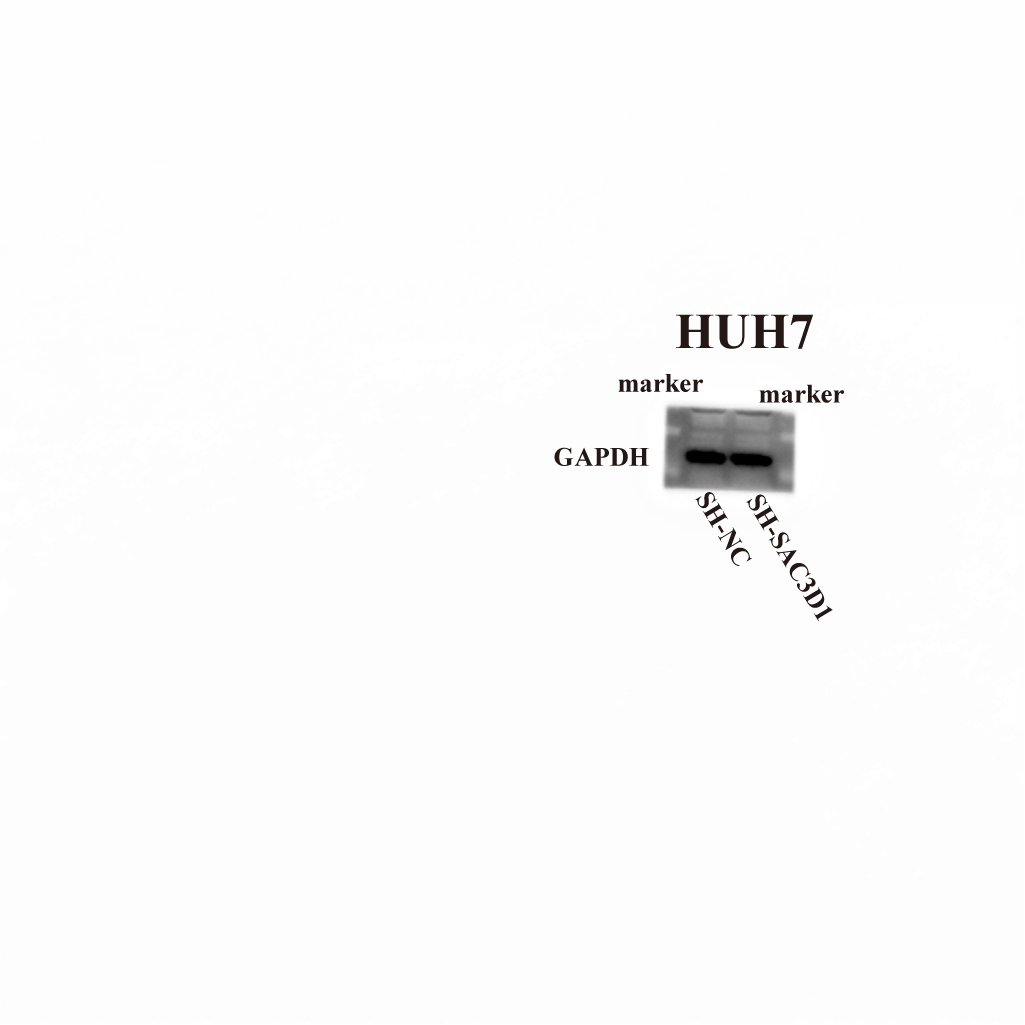

Supplement: Supplementary file 2 [file DataSheet2.ZIP › wb/GAPDH-HUH7-(3).tif]

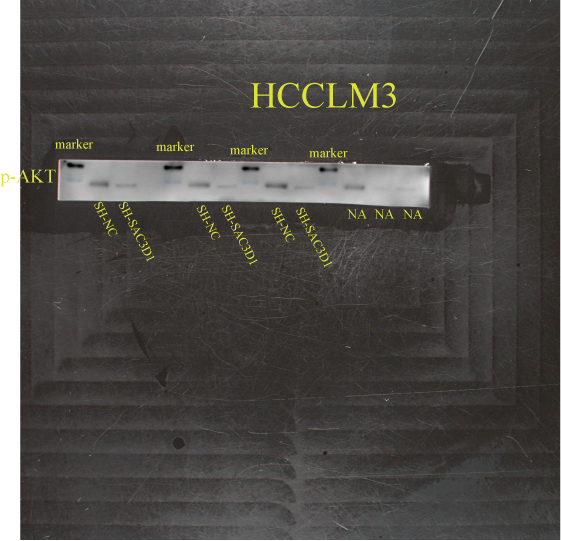

Supplement: Supplementary file 2 [file DataSheet2.ZIP › wb/p-AKT-HCCLM3-3 times.tif]

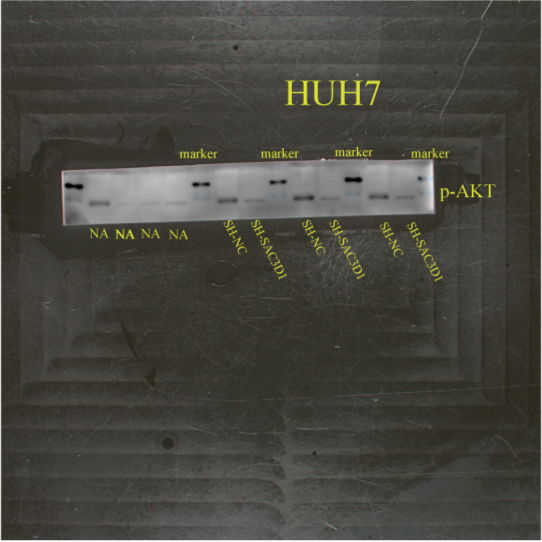

Supplement: Supplementary file 2 [file DataSheet2.ZIP › wb/p-AKT-HUH7-3 times.tif]

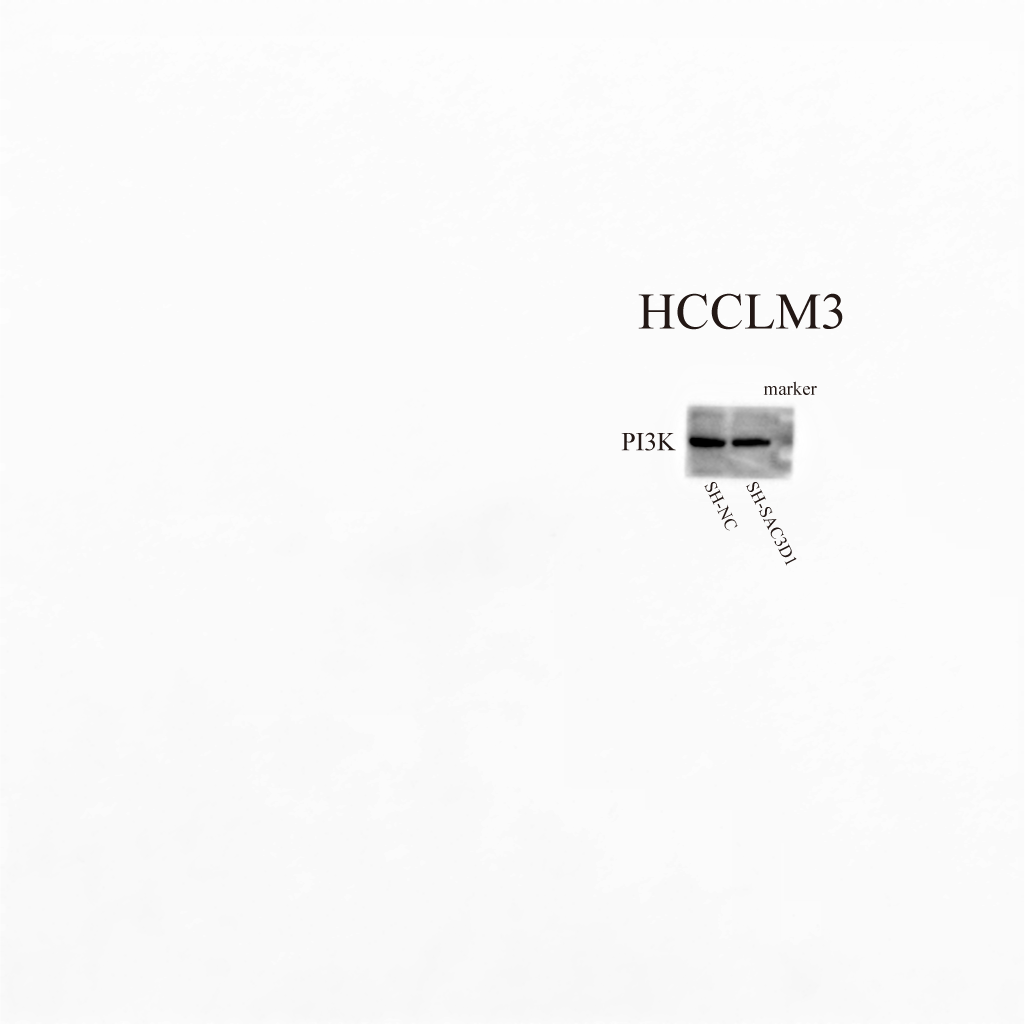

Supplement: Supplementary file 2 [file DataSheet2.ZIP › wb/PI3K-HCCLM3-(1).tif.tif]

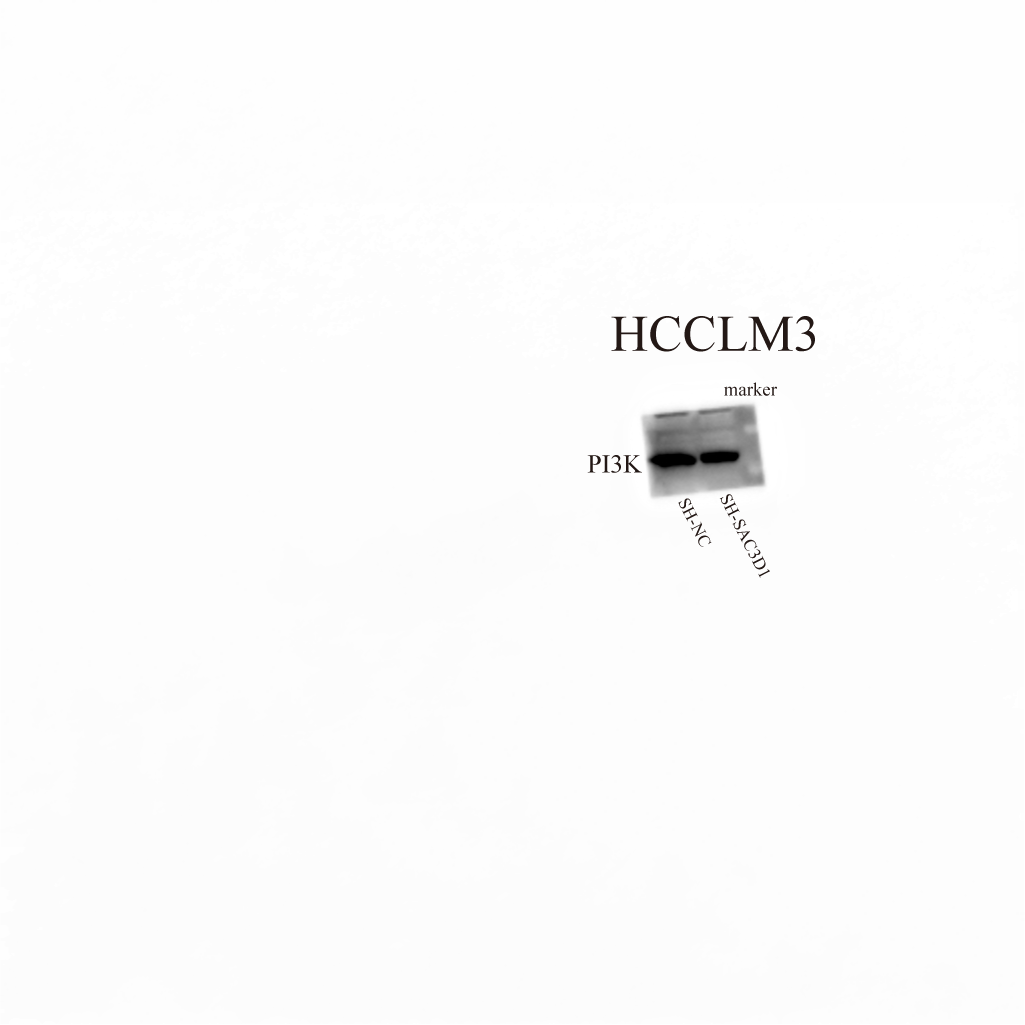

Supplement: Supplementary file 2 [file DataSheet2.ZIP › wb/PI3K-HCCLM3-(2).tif]

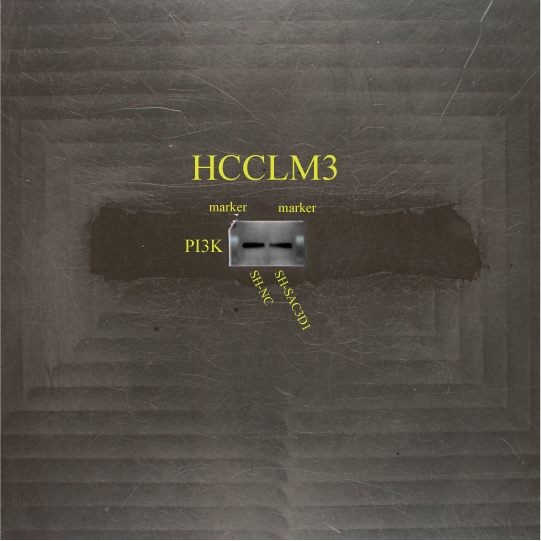

Supplement: Supplementary file 2 [file DataSheet2.ZIP › wb/PI3K-HCCLM3-(3).tif.tif]

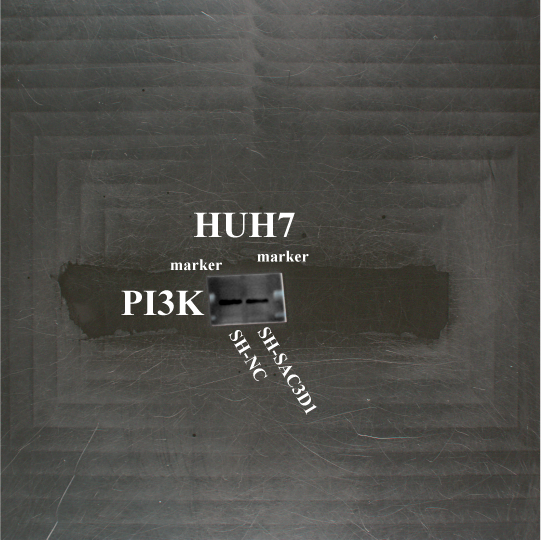

Supplement: Supplementary file 2 [file DataSheet2.ZIP › wb/PI3K-HUH7-(1).tif]

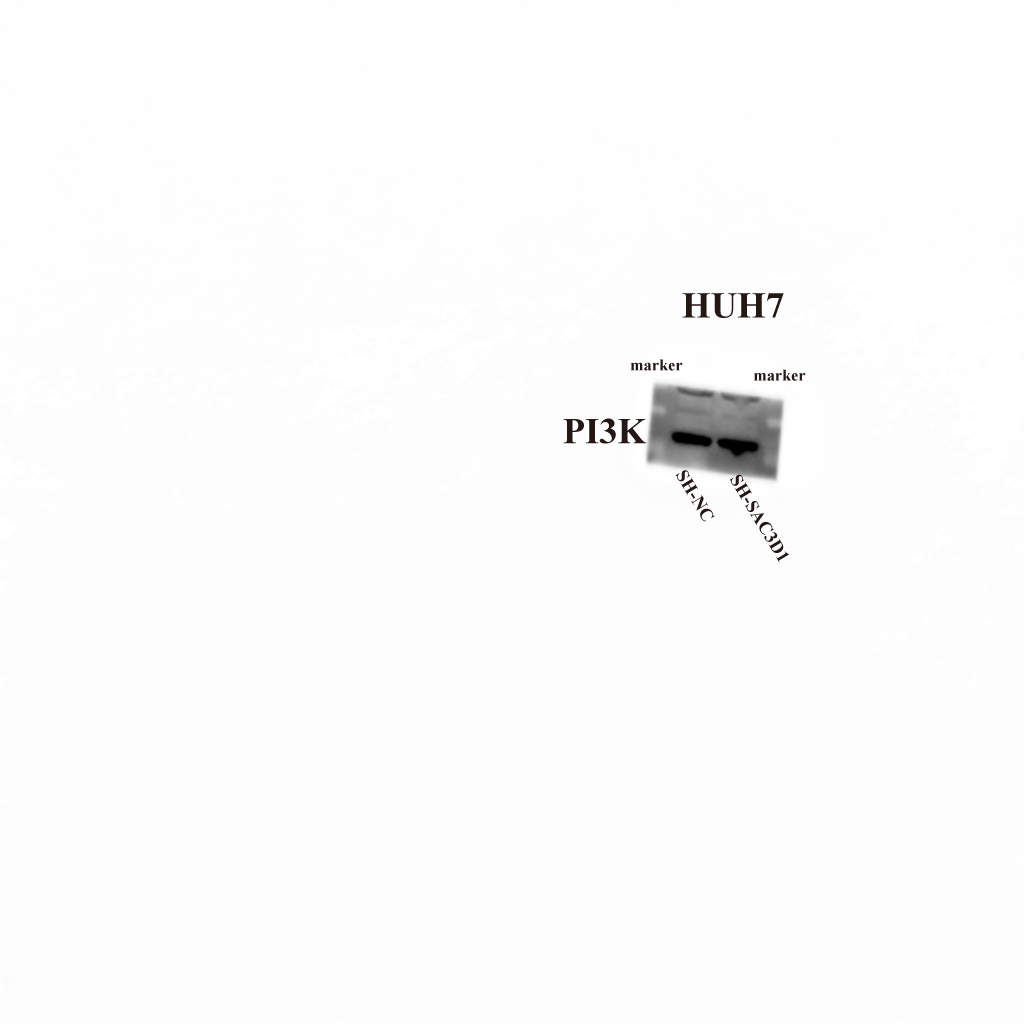

Supplement: Supplementary file 2 [file DataSheet2.ZIP › wb/PI3K-HUH7-(2).tif]

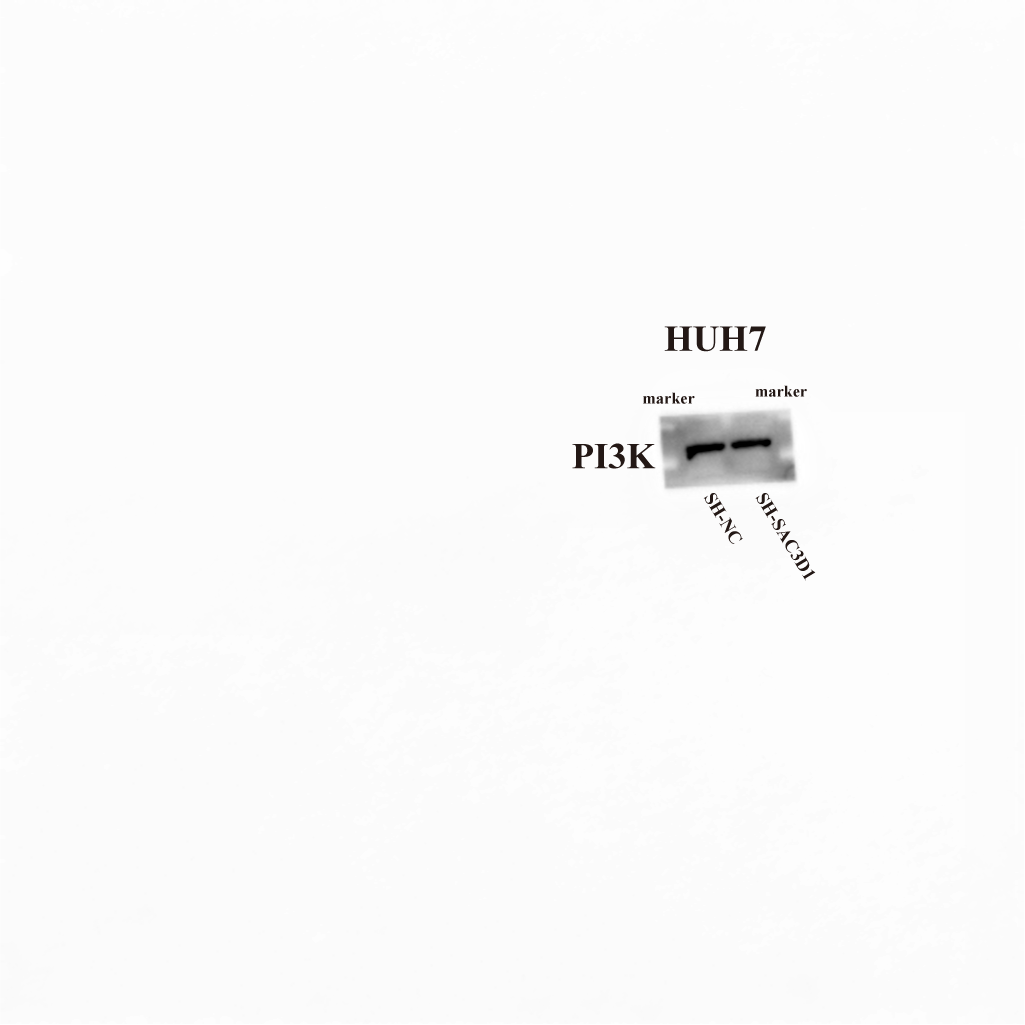

Supplement: Supplementary file 2 [file DataSheet2.ZIP › wb/PI3K-HUH7-(3).tif]

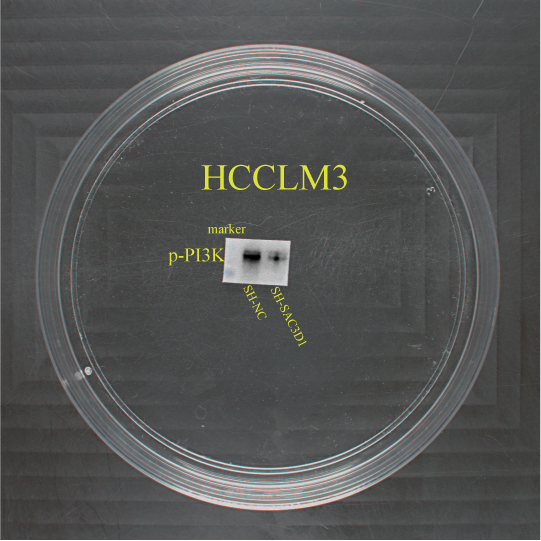

Supplement: Supplementary file 2 [file DataSheet2.ZIP › wb/p-PI3K-HCCLM3-ú¿1ú⌐.tif]

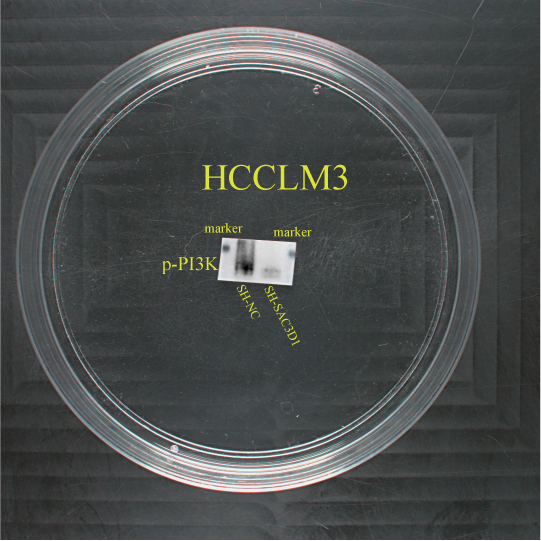

Supplement: Supplementary file 2 [file DataSheet2.ZIP › wb/p-PI3K-HCCLM3-ú¿2ú⌐.tif]

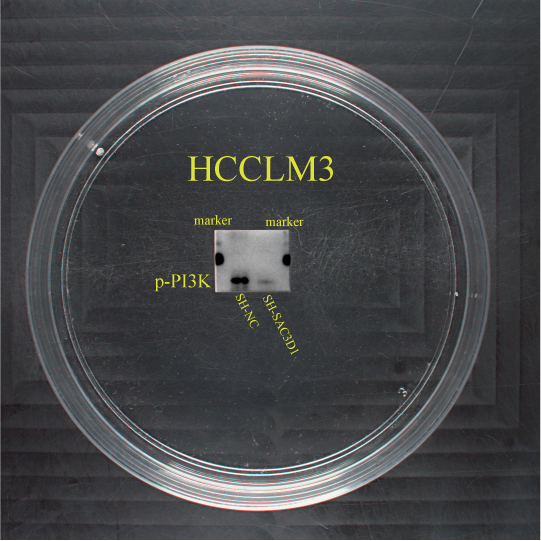

Supplement: Supplementary file 2 [file DataSheet2.ZIP › wb/p-PI3K-HCCLM3-ú¿3ú⌐.tif]

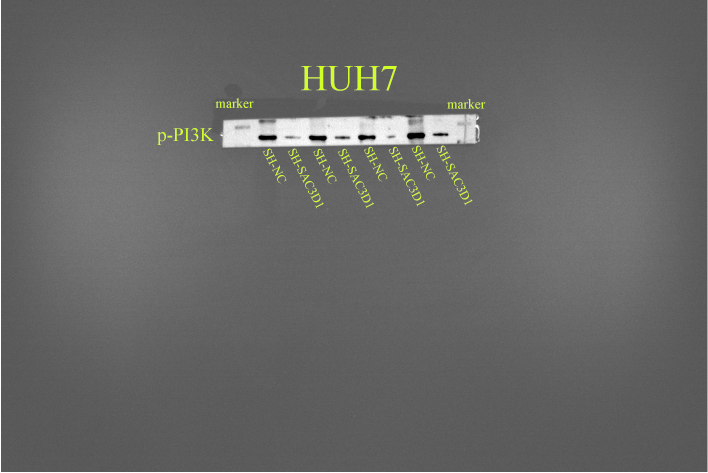

Supplement: Supplementary file 2 [file DataSheet2.ZIP › wb/p-PI3K-HUH7-3 times.tif]
